# Analysis of the hybrid genomes of two field isolates of the soil-borne fungal species *Verticillium longisporum*

**DOI:** 10.1186/s12864-017-4407-x

**Published:** 2018-01-03

**Authors:** Johan Fogelqvist, Georgios Tzelepis, Sarosh Bejai, Jonas Ilbäck, Arne Schwelm, Christina Dixelius

**Affiliations:** 1 Department of Plant Biology, Uppsala BioCenter, Linnean Center for Plant Biology, Swedish University of Agricultural Sciences, P.O. Box 7080, 75007 Uppsala, Sweden; 2 Present Address: National Food Agency, P.O. Box 622, 75126 Uppsala, Sweden

**Keywords:** *Brassica napus*, Carbohydrate active enzymes, Mating-type genes, *Verticillium longisporum*

## Abstract

**Background:**

*Brassica* plant species are attacked by a number of pathogens; among them, the ones with a soil-borne lifestyle have become increasingly important. Verticillium stem stripe caused by *Verticillium longisporum* is one example. This fungal species is thought to be of a hybrid origin, having a genome composed of combinations of lineages denominated A and D. In this study we report the draft genomes of 2 *V. longisporum* field isolates sequenced using the Illumina technology. Genomic characterization and lineage composition, followed by selected gene analysis to facilitate the comprehension of its genomic features and potential effector categories were performed.

**Results:**

The draft genomes of 2 *Verticillium longisporum* single spore isolates (VL1 and VL2) have an estimated ungapped size of about 70 Mb. The total number of protein encoding genes identified in VL1 was 20,793, whereas 21,072 gene models were predicted in VL2. The predicted genome size, gene contents, including the gene families coding for carbohydrate active enzymes were almost double the numbers found in *V. dahliae* and *V. albo-atrum*. Single nucleotide polymorphisms (SNPs) were frequently distributed in the two genomes but the distribution of heterozygosity and depth was not independent. Further analysis of potential parental lineages suggests that the *V. longisporum* genome is composed of two parts, A1 and D1, where A1 is more ancient than the parental lineage genome D1, the latter being more closer related to *V. dahliae*. Presence of the mating-type genes *MAT1–1-1* and *MAT1–2-1* in the *V. longisporum* genomes were confirmed. However, the *MAT* genes in *V. dahliae, V. albo-atrum* and *V. longisporum* have experienced extensive nucleotide changes at least partly explaining the present asexual nature of these fungal species.

**Conclusions:**

The established draft genome of *V. longisporum* is comparatively large compared to other studied ascomycete fungi. Consequently, high numbers of genes were predicted in the two *V. longisporum* genomes, among them many secreted proteins and carbohydrate active enzyme (CAZy) encoding genes. The genome is composed of two parts, where one lineage is more ancient than the part being more closely related to *V. dahliae*. Dissimilar mating-type sequences were identified indicating possible ancient hybridization events.

**Electronic supplementary material:**

The online version of this article (doi: 10.1186/s12864-017-4407-x) contains supplementary material, which is available to authorized users.

## Background

The *Verticillium* genus belongs to the *Plectosphaerellaceae* family in the *Sordariomycetes*, one of the largest classes in Ascomycota. The genus name derives from the “verticilliate” morphological feature of the branched conidiophore, the hyphal structure that bears conidia. *Verticillium* species incite vascular wilt diseases in many crops and wild plant species [1, 2]. No sexual stage has been reported so far that could help understanding speciation processes and adaptation to different ecological niches and range of plant hosts. *Verticillium longisporum* substantially shares the disease cycle characteristics with the more studied *V. dahliae*. As *V. longisporum* causes stunting but not wilting in infected plants it has been suggested to change Verticillium wilt to Verticillium stem stripe for the disease [3]. Further, the species name *“longisporum”* refers to the close to twice as long conidia found in most strains in comparison to *V. dahliae* [4–6]. This fungal species is known to have narrower host range compared to *V. dahliae* with preference for host species within the family *Brassicaceae*, including *Brassica* species and *Arabidopsis thaliana* [7, 8]. A recent study suggests a somewhat wider host-range [9]. In Sweden, sugar beet is not known as a host to *V. longisporum* but to *V. dahliae* [10].

Winter oilseed rape (*Brassica napus*) is the most important oil crop in Europe and the demand of products from this crop is increasing, not least in various new innovative and bioeconomical contexts. *V. longisporum* has become an important pathogen in most European countries, particularly in regions with intense oilseed rape production [3]. Spread of this plant pathogen is also reported from Canada (http://www.inspection.gc.ca/plants/plant-pests-invasive-species/diseases/verticillium-wilt/). A major problem is the longevity of its microsclerotia that are released into the soil from infected plant residues at the end of the disease cycle. These resting structures have the capacity to remain dormant in soil for many years. The disease cycle of *V. longisporum* is still unclear at certain parts but it is thought that the microsclerotia are stimulated to germinate via root exudates released from a host plant growing nearby. The hyphae then invade the lateral roots and root hairs followed by colonization of the root tissues and finally enter into the xylem elements. Next, conidia form and these spores can spread via the plant’s transpiration stream. Thus conidia are able to colonize vessel tissue further up in the plant, processes which could interfere with access to the xylem sap potentially affecting plant growth. Because the microsclerotia do not form and protrude stems and leaves until the plant is in the senescence phase, the disease infection easily remains unnoticed.

More than 50 years ago it was reported that some *Verticillium* strains had twice as long conidia and roughly double amount of nuclear DNA as those of *V. dahliae* [11]. Later, similar observations on the diploid status of a potentially new *Verticillium* species were published [4, 12]. Population genetic and phylogenetic studies supported that *V. longisporum* is a distinct species*.* This conclusion was based on restriction length fragment polymorphism (RLFP) and amplified fragment length polymorphism (AFLP) analyses, together with sequence data of mitochondrial and nuclear genes on a high number of fungal isolates [6, 13, 14]. The phylogenetic data indicated a closer relationship between *V. longisporum*, *V. dahliae* and *V. albo-atrum,* compared to *V. tricorpus* and the distantly related *V. nigrescens,* now placed in the *Gibellulopis* genus [15]. Further studies based on intron-rich sequences of five protein-encoding genes and ribosomal internal transcribed spacer sequences suggested that *V. longisporum* is a hybrid between *V. dahliae* and unknown lineages [16–18]. The progenitors were named A1 and D1, D2 and D3 all together resulting in 3 *V. longisporum* lineage compositions A1/D1, A1/D2 and A1/D3. The A1 and D1 progenitors were reported to be of unknown origin whereas D2 and D3 were suggested to represent 2 *V. dahliae* lineages. For further background and details on this fungus, see reviews by Depotter et al. [3, 19].

In the present study we aimed to establish new information on the *V. longisporum* genome composition by sequencing two fungal strains (VL1 and VL2), and to compare the data with genomes from three other *Verticillium* species; *V. dahliae*, *V. albo-atrum* and *V. tricorpus* [20, 21].

## Results

### Mapping and SNP analysis

First, Illumina sequence data generated from DNA of the 2 *V. longisporum* strains were mapped to the PacBio sequences of *V. dahliae* [22]. Most of the *V. longisporum* paired-end reads could be consistently mapped to the *V. dahliae* reference genome (86 and 79% in VL1 and VL2, respectively) and most of the *V. dahliae* reference genome was covered by *V. longisporum* reads, 93% (VL1) and 94% (VL2). The distribution of coverage was bimodal in both *V. longisporum* strains, representing reads originating from one or both parental genomes, with peaks at 100× and 200× for VL1 and 75× and 150× for VL2 (Additional file 1). Single nucleotide polymorphisms (SNPs) were distributed, on average one in 24.37 bp (VL1) and one in 23.81 bp (VL2), in total 1,404,670 and 1,438,074 SNPs, respectively. The vast majority of SNPs was heterozygous, 84.7% in VL1 and 84.6% in VL2. The distribution of heterozygosity and depth was not independent, as expected considering a hybrid genomic constitution of this species. The heterozygosity of SNPs with a depth of coverage corresponding the largest peak in the bimodal coverage distribution was about 0.92 in both VL1 and VL2, whereas SNPs with a depth corresponding to the smaller peak had a heterozygosity of about 0.5 (Additional file 1).

### Evolution of the *V. longisporum* genome

We looked at the parental lineage origin at the coding sequence level in our data sets because of the suggested composition of A and D sub-genomes in the genome of *V. longisporum* [16]. We successfully reconstructed 639 and 668 regions in VL1 and VL2, with an average length of 29.0 ± 17.4 and 29.3 ± 18.7 kb. In total 6471 (VL1) and 6788 (VL2) transcripts were successfully phased, which is 56 and 59% of the total number of transcripts in *V. dahliae*. The distribution of the fourfold degenerate transversion (4DTv)-rate was different when comparing *V. dahliae* to each of the parental genomes of VL1 and VL2. For both VL1 and VL2 there was a clear peak at 4DTv = 0.006 in the comparison between *V. dahliae* and the D parental genome, whereas there was a peak at 4DTv = 0.05 for the comparison between VD and the A parental genome as well as for comparing the A to the D parental genome (Additional file 2). When comparing the A and D between VL1 and VL2 there were peaks at 4DTv = 0.003 for both parental genomes. This suggests a close relationship between *V. dahliae* and the D parental genome. Further, from the collinear block analysis among the genomes of *V. dahliae* and *V. albo-atrum* (and the outgroup *V. tricorpus*)*,* in total 7244 genes could unambiguously be assigned as orthologous, present in exactly one copy in each genome. Successfully reconstructed parental origin in both VL1 and VL2 were possible for 3592 genes. The analysis of 4DTv suggested that *V. albo-atrum* was slightly closer to A than to D, with peaks at about 4DTv = 0.08 and 0.09, respectively (Additional file 2). When we constructed a phylogenetic tree using a concatenation of these genes (3592), *V. longisporum* D was placed close to *V. dahliae* (Additional file 3). Together with *V. albo-atrum* these three genomes formed a monophyletic clade with *V. longisporum* A as a sister group. Whereas the bootstrap values were overall high, gene support frequency and internode certainty values were low for this clade.

### Genome characteristics

The de novo draft genome sequences achieved of the VL1 and VL2 strains consisted of 4620 (VL1) and 6431 (VL2) scaffolds over 500 bp with N_50_ values of 154,661 (VL1) and 91,201 (VL2) (Table 1). Different assemblers were tried, for example Redundans [23] but no significant improvement in reducing scaffold numbers was achieved compared to the chosen approach. Further, an un-gapped assembly size would reduce the genome size from 95 Mb to 70 Mb. Evaluation based of the CEGMA analysis revealed that 94% (VL1) and 76% (VL2) of the eukaryotic core genes were full length (98 and 96% partial) indicating a fragmented assembly. The GC content of the two genomes was 55.8% (VL1) and 53.3% (VL2), and the mitochondrial genome sizes were estimated at 27.7 kb and 26.2 kb, respectively. The total number of protein encoding genes identified in VL1 was 20,793, and 21,072 gene models were predicted in VL2. The majority (13,334 and 12,728) of the predicted gene models could be partitioned into syntenic blocks with *V. dahliae* and/or *V. albo-atrum*.

**Table 1:** Description of genome assemblies of the 2 *V. longisporum* isolates VL1 and VL2

		VL1	VL2
Nuclear genome	Scaffold length (Mb)	~95	~95
	Genomic GC content (%)	55.84	53.34
	Scaffold number	4620	6431
	Scaffold N50	154,651	91,201
	Scaffold L50	150	221
	Contig N50	26,946	73,321
	Contig L50	710	270
	Number of contigs	14,286	13,586
	Contig length (bp)	67,181,795	67,661,737
	Total number of genes	20,793	21,072
Mitochondrial genome	Scaffold length (bp)	27, 669	26,151
	GC content (%)	27.13	27.82
	Scaffold number	1	1
	Number of contigs	3	2
	Contig length (bp)	26,438	26,116

Based on intron-rich sequences of: actin, elongation factor 1-alpha, glyceraldehyde-3-phosphate dehydrogenase, mitochondrial oxaloacetate transport protein and tryptophan synthase gene sequences, *V. longisporum* was suggested to be a diploid fungal species generated by hybridization between three different D lineages with difference in phylogenetic distance to *V. dahliae*, and the hypothetical ancestor species A1 [16]. By using these five gene sequences including ribosomal internal transcribed spacer (ITS) sequences together with the most similar sequence from VL1 and VL2, we conclude based on phylogenetic reconstruction (Additional file 4A–F) that both our fungal strains were of the A1/D1 type using the nomenclature by Inderbitzin and co-workers [3].

Despite the relatively high numbers of scaffolds achieved, the repeat content in the 2 *V. longisporum* genomes was low, ranging between 3.13 to 7.87% of unclassified repeats, and 1.35 to 3.04% of identified transposable elements (TEs) (Additional file 5). This low level of TEs in *V. longisporum* is consistent with the percentages found in *V. dahliae* and *V. albo-atrum* [20, 24]. To further decipher the potential role of retrotransposons in genomic rearrangements, we performed a combination of RepeatMasker searches and manual inspections of our datasets. Among the retrotransposons identified, the most abundant group was characterized as long terminal repeats (LTR) belonging to class I (Additional file 5). *LINE1* was completely absent in VL1, but present in VL2 based on our data. In contrast, VL2 harbored a higher number of *LTR/Gypsy* elements, 4402 compared to 1338 in VL1. DNA transposons categorized as class II were also more frequent in the VL2 genome, and *hAT-Ac*, *TcMar-Pogo* and *PIF-Harbinger* were all present in VL2 but absent in VL1. *Copia*, *Gypsy* and *Tc1/Mariner* are the most widespread transposons in *V. dahliae* and *V. albo-atrum,* known to accumulate in gene-rich regions or in dispersed chromosomal spots [25]. No biased locations of TEs in the 2 *V. longisporum* genomes were however detected in our dataset.

### Mating-type genes

It is known that mating type in Ascomycota is highly variable and consist of dissimilar sequences or idiomorphs [26]. The *MAT1–1-1* gene harbors the α1 domain while the *MAT1–2-1* gene encodes a transcription factor with a MATA_HMG domain. Due to the hybrid nature of the *V. longisporum* genome we searched for mating type information among the *Verticillium* species. *MAT1–2-1* is annotated in the *V. dahliae* reference genome as VDAG_JR2_Chr2g11860. In contrast, the other idiomorph characterized by the α1-box containing *MAT1–1-1* gene is lacking but present in Genbank. *MAT-1-1-1* could be identified in *V. albo-atrum* by BLAST search (VDBG_01985T0, E-value 0.0) but no *MAT1–2-1* sequence is available.

**Fig. 1:**
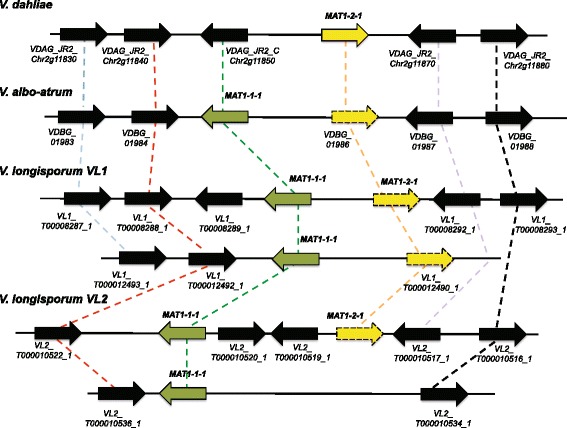
Genomic organization of mating type genes, in three *Verticillium* species. In the *V. longisporum* genome sequences the mating type genes were identified on two different contigs in both VL strains. *MAT1–1-1* (green), *MAT1–2-1* (yellow) and neighboring genes (black). Dashed lines in different colors (blue, red, green, yellow, orange, purple and black) indicate homology among the genes. VDAG_JR2_Chr2g11850 (black) lacks the HMG-box conserved in other MAT1–1-1 proteins. Details on PFAM predictions and levels of sequence homologies are listed in Additional file 6. Gene ID is indicted. The illustration is not drawn to scale

**Fig. 2:**
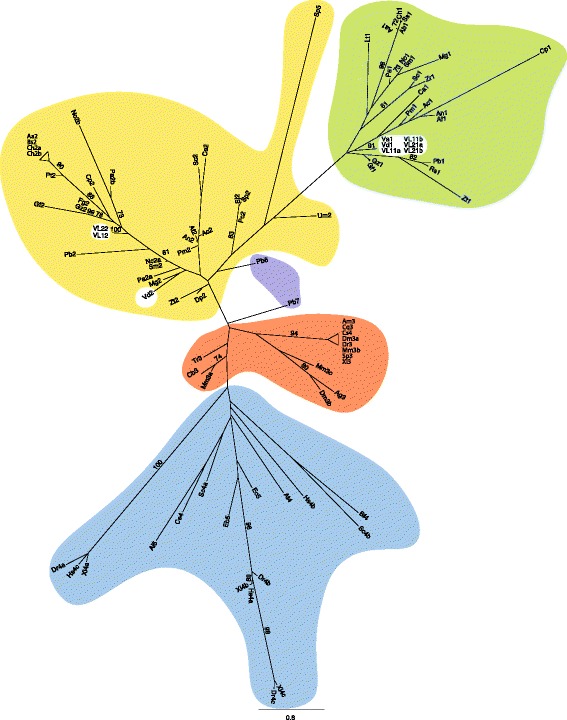
Maximum likelihood phylogeny (RAXML, model LG+Γ) of the HGM superfamily and the α1 domain core amino acid sequence integrated in a slightly modified dataset [22] now incorporating mating type genes found in *V. longisporum* strain VL1 and VL2, and *V. albo-atrum*. Bootstrap support values above 70 are shown. Labeling is as follows: α1 (green), MATA_HMG (yellow), SOX (orange), HMGB (blue), *Phycomyces blakesleeanus* (Zygomycota), sexM and sexP are circled in purple and *Verticillium* species (white). The number after the abbreviation indicates the domain as: 1, α1; 2, MATA_HMG; 3, SOX; 4, HMGB; 5, HMG; 6, SeqP and 7, SeqM. In the case more than one domain is present in a species, the suffix a, b, c is added. Accession numbers for proteins used for the phylogeny is listed in Additional file 14. Species abbreviations: Aa, *Alternaria alternata*; Ab, *Alternaria brassicicola*; Ac, ı; Af, *Aspergillus fumigatus*; Ag, *Anopheles gambiae*; Al, *Antonospora locustae*; Am, *Ailuropoda melanoleuca*; An, *Aspergillus nidulans*; At, *Arabidopsis thaliana*; Bf, *Botryotinia fuckeliana*; Bs, *Bipolaris sacchari*; Ca, *Candida albicans*; Cb, *Cervus elaphus yarkandensis*; Ce, *Caenorhabditis elegans*; Ch, *Cochliobolus heterostrophus*; Cp, *Cryphonectria parasitica*; Cq, *Culex quinquefasciatus*; Cs, *Ciona savignyi*; Dm, *Drosophila melanogaster*; Dp, *Dothistroma pini*; Dr., *Danio rerio*; Eb, *Enterocytozoon bieneusi*; Ec, *Encephalitozoon cuniculi*; Fg, *Fusarium acaciae-mearnsii*; Gf, *Gibberella fujikuroi*; Gz, *Gibberella zeae*; Hs, *Homo sapiens*; Lt, *Lachancea thermotolerans*; Mg, *Magnaporte grisea*; Mm, *Mus musculus*; Nc, *Neurospora crassa*; Pa, *Podospora anserina*; Pb, *Pyrenopeziza brassicae*; Pc, *Pneumocystis carinii*; Pm, *Penicillium marneffei*; Pt, *Pyrenophora teres*; Rs, *Rhynchosporium secalis*; Sc, *Saccharomyces cerevisiae*; Sj, *Schizosaccharomyces japonicus*; Sm, *Sordaria macrospora*; Sp, *Schizosaccharomyces pombe*; Ss, *Stemphylium sarciniforme*; Tr, *Takifugu rubripes*; Um, *Ustilago maydis*; Va, *Verticillium albo-atrum*; Vd, *Verticillium dahliae*; VL1, *Verticillium longisporum*; VL2, *Verticillium longisporum*; Xl, *Xenopus laevis*; Zr, *Zygosaccharomyces rouxii*; Zt, *Zymoseptoria tritici*

Searching for homologous genes in our *V. longisporum* genome data revealed two copies of *MAT1–1-1* and one copy of *MAT1–2-1* in both VL1 and VL2 strains. Typically the two alternate sequences should occupy the same locus on corresponding chromosomes [27]. Here, the *MAT1–2-1* sequence was located next to one of the *MAT1–1-1* genes in both *V. longisporum* strains, whereas the other copy of *MAT1–1-1* was found on a separate contig (Fig. 1). The *Verticillium* mating type sequences were integrated in a slightly modified multi-species dataset earlier used for the reconstruction of the phylogenetic relationship of the HMG and α1 domain sequences [28]. All *MAT1–1-1* and *MAT1–2-1 Verticillium* domain sequences grouped together with other sequences from Pezizomycotina in Ascomycota (Fig. 2). The single VL *MAT1–1-1* copy is more closely related to the *V. dahliae* gene copy (green sub-clade) and probably derived from the D genome, whereas the VL copy of *MAT1–2-1* (yellow sub-clade) is more distant to the *V. dahliae* sequence and by analogy should have derived from the A genome. The presence of *MAT1–1-1* and *MAT1–2-1* genes in the genome of both *V. longisporum* isolates was confirmed by PCR amplification followed by Sanger sequencing. When more closely analyzing and comparing the complete *MAT* sequences, and flanking genes, a more complex picture appeared. Overall synteny can be seen but rearrangements have occurred in these chromosomal regions including deletions and inversion/insertion events (Fig. 1). Sequence similarity data further suggests that the *MAT* genes have been exposed to rather extensive changes. When aligning the sequences using the Pfam database, a loss of the HMG box regulating mating in VDAG_7R2_Chr2g11850 was revealed most likely leading to loss of function in this gene (Additional file 6).

### Secretome analysis

Using a combination of bioinformatics tools, we identified a total of 1281 and 1251 secreted proteins in VL1 and VL2, respectively. This number is higher compared to *V. dahliae* (746) and *V. albo-atrum* (767) but slightly less if the hybrid nature of *V. longisporum* is considered (Additional file 7). Eukaryotic orthologous groups (KOG) analysis of the secreted proteins revealed approximately twice as many proteins involved in ion transport and metabolism, lipid transport and metabolism in VL1 compared to *V. dahliae* and *V. albo-atrum* (Additional file 8). Pathogens secrete a variety of proteins that include potential pathogenicity factors, generally named as effectors [29]. These effectors commonly cysteine-rich proteins are known to suppress or interfere with immune responses of the host metabolism to facilitate pathogen colonization. All the predicted secreted proteins in the 2 *V. longisporum* strains possessed highly variable numbers of cysteine (Additional files 9, 10 and 11). Both *V. longisporum* genomes were found to harbor fourteen secreted proteins containing a cysteine-rich fungal-specific extracellular EGF-like (CFEM) domain. Four EGF-like gene copies are present in each of the genomes of *V. dahliae* and *V. albo-atrum* [20] but the function of these genes are not understood. By exploiting the proteins predicted as secreted in *V. longisporum*, we were able to detect 204 (VL1) and 203 (VL2) potential effector candidates (Additional file 7). Among these cysteine-rich proteins more than 50% varied between 200 and 300 amino acids in size. The number of pairwise distances < than 10 kb was higher than expected among the candidate effectors in both VL1 and VL2 genomes. The effector candidate sequences were clustered to some degree (Additional file 12). The number of pairwise distances among secreted proteins was enriched in the interval 0–20 kb for VL1 and in the interval of 10–20 kb in VL2.

Conserved secreted proteins play a significant role for fungal pathogens, and genes coding for LysM effectors residing in the CBM50 peptidoglycan-binding module, and necrosis- and ethylene-inducing-like proteins (NLP) were previously shown to contribute to *V. dahliae* pathogenicity [24, 30]. Our *V. longisporum* genomes were predicted to host eleven (VL1) and eight (VL2) genes harboring the LysM motif (Additional file 7). NLP-encoding genes are reported to be present in most fungal genomes, and possess cytotoxic activity towards many plant species [31]. In our analysis, we found twelve and fifteen gene homologs in VL1 and VL2 respectively with the necrosis-inducing *Phytophthora* protein or NPP1-domain, compared to eight in *V. dahliae* and seven in *V. albo-atrum*.

### Carbohydrate active-enzymes

**Fig. 3:**
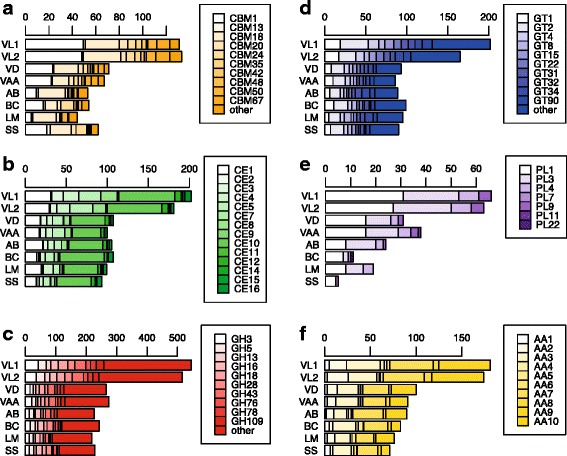
Total numbers of CAZy families and domains in *V. longisporum* (VL1 and VL2), and selected plant pathogenic fungi. VD = *V. dahliae*, VAA = *V. albo-atrum*, AB = *Alternaria brassicicola*, BC = *Botrytis cinerea*. LM = *Leptosphaeria maculans*, SS = *Sclerotinia sclerotiorum.* (**a**) CAZy families with carbohydrate binding domains (CBM), (**b**) carbohydrate esterases (CE), (**c**) glycosyl hydrolases (GH), (**d**) glycosyl transferases (GT), (**e**) pectate lyases (PL), and (**f**) auxiliary activities (AA). To the right, lists of individual CAZy domains

Several studies have demonstrated a strong relationship between the carbohydrate active enzyme (CAZy) repertoire in fungal genomes and their saprophytic, parasitic or necrotrophic life-style strategies [32, 33]. The analyses of the two *V. longisporum* genomes revealed a rich repertoire of CAZy families, with close to twice as many when compared to *V. dahliae*, *V. albo-atrum*, and four fungal pathogens known to incite disease on *Brassica* crops: *Alternaria brassicicola*, *Botrytis cinerea*, *Leptosphaeria maculans* and *Sclerotinia sclerotiorum* (Fig. 3; Additional file 13). Among the potential effectors (<400 amino acids) in the *V. longisporum* genomes, thirteen (VL1) and ten (VL2) contained fungal cellulose-binding domains. The second major sub-group in VL1 and VL2 belonged to the glycosyl hydrolase family with twelve (VL1) and eleven (VL2) proteins. The *V. dahliae* and *V. albo-atrum* genomes are enriched for polysaccharide lyases compared to other sequenced ascomycete fungi [20]. Here we found only ten and nine pectate lyases, respectively, in the two VL genomes with potential effector characteristics (Additional files 9 and 10).

## Discussion

*Sordariomycetes* (former *Pyrenomycetes*) is one of the largest classes in the Ascomycota comprising diverse species to which *V. longisporum* belongs [3]. Among others, members of this fungal class are known to cause diseases and produce many important secondary metabolites, some with toxic consequences for animals and humans [34–37]. The family *Plectosphaerellaceae* harbors a handful of other plant pathogens than *Verticillium* species, for example the legume pathogen *Volutella colletotrichoides* that is placed in a related genus [38]. The *Verticillium* genus has historically harbored a wide range of plant parasites and saprotrophs but has undergone several taxonomic revisions excluding all species except a handful of fungal plant pathogens.

The list of fungal genomes sequenced and publicity available is constantly growing. The fungal genome sizes are presently estimated to range between 8.97 Mb to 117.57 Mb, all with high gene diversity and content of repetitive DNA and TEs [39]. The *V. dahliae* and *V. albo-atrum* genomes have predicted sizes of 35 Mb and 30 Mb, respectively, [20] and the present draft genome of *V. longisporum* has about double this size, which is in line with its proposed hybrid nature. A major force in evolution is genome duplication sometimes involving hybridization, which together are important features of the evolutionary history in many eukaryotic organism groups. Increasing numbers of hybrid species in fungi are described particularly in species such as *Saccharomyces*, *Pichia*, *Cryptococcu*s, and *Candida* [40–44]. Likewise, among plant pathogens, hybridization is increasingly being recognized as an important evolutionary factor [19].

Based on earlier AFLP studies on the European *V. longisporum* population we can conclude that Swedish and German *V. longisporum* isolates from oilseed rape are very similar [13]. In a wider comparison of European *V. longisporum* isolates using microsatellites or simple sequence-repeat markers, Swedish and German isolates are referred to the eastern subgroup of the A1/D1 genome composition [45]. In analogy and based on new data reported here we assign the D1 lineage as having a *V. dahliae* progenitor. In our efforts to generate more information on the ancestor generating lineage A1 we estimated the distribution of synonymous substitution rate (Ks) between *V. dahliae* and the *V. logisporum* orthologous genes. The modal values of Ks in the *V. dahliae* to *V. longisporum* orthologous genes with *V. dahliae* origin were close to 0.025 but close to 0.1 for the genes with the hypothetical ancestor origin. For all other comparisons (*V. dahliae* to *V. albo-atrum*, *V. albo-atrum* to *V. longisporum*) the modal values of Ks were close to 0.15. Assuming a neutral mutation rate of 1 × 10^−9^ per site and year [46] these Ks values suggest that divergence between *V. dahliae* and *V. albo-atrum* occurred about 75 million years ago (Myr). The divergence time between *V. dahliae* and *V. longisporum* have two parts; a divergence from the part with the hypothetical ancestor lineage A1 about 50 Myr ago and divergence from the assumed *V. dahliae* parent (D1) about 12.5 Myr ago. Hitherto we have not been able to find any sequence information that could reveal the ancestry of the lineage A1.

Hybridization events have played exceptional important roles for speciation in the host family *Brassicaceae* [47], which experienced ancient whole-genome duplication about 47 Myr ago [48] followed by losses of duplicated genes. Several splits and genome duplications have followed forming three lineages where today’s species in this plant family can be placed. *V. longipsorum* infects species in lineage I (e.g. Arabidopsis) and lineage II (Brassica) whereas information on diseased plants from lineage III is lacking. It is premature at this stage to suggest co-evolution of ancestor *Verticillium* species with their host plants particularly when no sexual stage is known that could accelerate such adaptation. However, it has been suggested that the A1 lineage derive from species that used plant species in *Brassicaceae* as a host [19]. Notably, the first report of *V. longisporum* as a plant pathogen on *Brassica* crops was as recent as 1970 [49]. *V. longisporum* is now reported from many countries, thus the presence of different ancestor species and their fusion with potentially different *V. dahliae* isolates are not unlikely but the question on what factors that have triggered such events in this case remains unanswered. In a survey of a global collection containing 1120 *V. dahliae* isolates, as few as 12 contained the *MAT1–1* idomorph whereas 1108 had the *MAT1–2* [50]. With this fragmentary knowledge, we propose that host adaptation in case of *V*. *longisporum* is a later event separate from the speciation process and that the sexual mode of reproduction has gone lost in the meantime. The latter switch only requires a single nucleotide change within a *MAT* locus [51]. Further, presence and function of transcript regulators and pheromone-associated genes required for mating is another knowledge gap among species in the *Verticillium* genus.

The genomes of the vascular colonizing fungi *V. dahliae* and *V. albo-atrum* are enriched in carbohydrate modifying enzymes, particularly within the glycoside hydrolases (GH) group [20]. Their function is multifaceted, and could for example include release of cell wall carbohydrates as nutrient sources for fungal metabolism due to the hydrolysis of the glycosidic bond between carbohydrate(s) and non-carbohydrate moieties [52]. The secreted protein VdSSP1 from *V. dahliae* has been previously shown to be involved in degradation of certain plant cell-wall compounds [53], and we anticipate a similar function for several GH enzymes of *V. longisporum*. Many of these carbohydrate modifying enzymes are encoded in the *V. longisporum* genome but their functions are not known.

## Conclusions

In this study, DNA from two single spore isolates of *V. longisporum* VL1 and VL2 were sequenced using the Illumina technology. The predicted ungapped genome size and the number of predicted protein coding genes is about double of the numbers generated from the *V. dahliae* and *V. albo-atrum* genomes [20]. A large proportion of the predicted gene models in *V. longisporum* could be partitioned into syntenic blocks with *V. dahliae* and/or *V. albo-atrum*. A hybrid genomic feature of *V. longisporum* was supported by SNP analysis. Searching for homologous genes in our VL genome data revealed two copies of *MAT1–1-1* and one copy of *MAT1–2-1* in both VL1 and VL2 strains encoding either the α1-box or the MATA_HMG domains. Extensive sequence changes have occurred in these mating genes suggesting a rather ancient descent, further supported by the overall distribution of synonymous substitution rate (Ks) between *V. dahliae, V. albo-atrum* and the *V. longisporum* orthologous genes. The two VL genomes analyzed in this work could be assigned to the A1/D1 nomenclature of the hybrid genome with the ancestor lineage deriving from *V. dahliae* in the D1-part, whereas the nature of A1 is unknown. Many questions remain to be answered on the *V. longisporum* genome evolution, and its host interactions. Overall, the genomic study reported here provides an important milestone of this important pathogen on *Brassica* crops.

## Methods

### Fungal isolates and growth conditions

The single spore isolates VL1 (CBS110220) and 43–3 from the culture collection at the Plant Biology Department, Swedish University of Agricultural Sciences (here denoted VL2), determined as *V. longisporum* by RLFP [6] and AFLP analysis [13] were used in the study. The isolates derived from diseased *Brassica napus* plants taken at different sites in southern Sweden, and have a modest polymorphism difference based on RFLP analysis [6]. The nuclear content of VL1 was 0.065 pg DNA and 0.051 pg DNA in VL2, determined by flow cytometry [6]. Culture and storage of the isolates was as described earlier [6]. Fungal mycelia were used as the source for the DNA and RNA preparations.

### Fungal DNA and RNA preparation

Total DNA from *V. longisporum* was extracted with Fermentas GeneJET Plant Genomic DNA Purification Mini kit (Thermo Fisher Scientific). RNA was isolated using GeneJET RNA purification kit (Thermo Fisher Scientific). Quality controls for RNA and DNA were carried out using Bioanalyzer (Agilent Techn.).

### Genome sequencing

Genomic DNA was sequenced with Illumina technology and filtered from adaptor sequences and low quality reads at BGI Hong Kong (China). For each fungal sample a paired-end (200 bp insert size) and a mate-pair (5 kb insert size) library were used. In total 7.48 and 7.50 Gbp high quality data were generated for VL1 and VL2, respectively. All reads were error corrected using Quake v0.3.4 [54].

### Mapping

First, the Illumina reads from VL1 and VL2 were mapped to the *V. dahliae* reference genome (GCA_000400815.2), based on strain JR2 [22] using BWA-MEM v0.7.8 [55] with default settings. Variants were called jointly using Freebayes v0.9.21 (−-use-best-n-alleles 5 -p 2 --hwe-priors-off --haplotype-length 0) [56] and filtered using GATK VarFiltration v3.3.0 (−filter “QUAL <20.0” -filterName SAP -filter ″SAP.0 > 30.0 & & SAP.1 > 30.0) [57]. Assuming that *V. longisporum* is a hybrid between *V. dahliae* and some other *Verticillium* species [3], we made an effort to differentiate between the variants originating from the different parental lineages. The mapped reads were phased using Samtools v0.1.19 (https://sourceforge.net/projects/samtools/files/samtools/0.1.19/), resulting for each *V. longisporum* lineage, a set of phased regions. Each phased region was furthermore divided whenever there were variants that could not be unambiguously resolved. Variants were called for the four different mapped set of *V. longisporum* reads jointly using Freebayes v0.9.21 (−-use-best-n-alleles 5 -p 2 --hwe-priors-off --haplotype-length 0) as described [58] and filtered using GATK VarFiltration v3.3.0 (−filter “QUAL <20.0” -filterName SAP -filter ″SAP.0 > 30.0 & & SAP.1 > 30.0). Only homozygous single nucleotide polymorphisms (SNPs) were considered from here on. For each phased region and *V. longisporum* strain, the overlapping transcripts were extracted, and modified by replacing the nucleotides in the polymorphic sites with the appropriate base for each phase. Transcripts containing internal stop codons and phased regions containing less than five transcripts were excluded from further analysis. The four-fold degenerate transversion rate (4DTv) between each phased *V. longisporum* transcript and the *V. dahliae* transcript was calculated using SeqinrRv3.3.1 [59]. Only regions where at least 60% of the transcripts in one phase had a larger 4DTv rate than in the other phase was further analyzed. This was the case for >75% of the regions in both VL1 and VL2. Collinear blocks among *V. dahliae*, *V. albo-atrum* [20] and *V. tricorpus* [21] were identified using MCScanX [60] under default settings. The 4DTv-rate was calculated for all identified pairs of coding genes. The alignment was based on Muscle v3.8.31, and protein sequences [61] within identified collinear blocks. Whenever a comparison was made to *V. dahliae*, it was also made to VL1 and VL2 if that gene was among the set of successfully phased genes that could be partitioned into parental genomes. A maximum likelihood phylogenetic tree was created using RaXML v8.9.2 on the concatenated alignment using GTR-Γ [62], random starting tree, rapid bootstrapping (1000 replicates) and using *V. tricorpus* as outgroup. For calculation of gene support frequency and internodal certainty gene trees were likewise generated, using the same settings but automatic bootstrap criteria (autoMRE).

### Assembly

A number of assembly approaches were tried. In the end, the best de novo assemblies were generated using Velvet v1.2.9 [63] with a kmer size of 49 bp and kmer coverage cutoff at 10×. The assemblies were further scaffolded using SSPACE v2.0 [64] with default settings and gap-filled using GapFiller v1.11 [65]. Mitochondrial sequences were manually identified by their coverage, GC-content, and similarity to *V. dahliae* and *V. albo-atrum* mitochondria [20]. The completeness of the two genomes was assessed by mapping a set of 248 core eukaryotic genes to the de novo assembly using CEGMA [66]. Transcriptome data were generated by Illumina paired-end strand-specific sequencing of the mRNA libraries at SciLife Lab, Stockholm (Sweden). Libraries were prepared by the sequencing platform under their in-house conditions. After quality filtering using ConDeTri v2.2 [67] 113.8 and 84.08 millions high quality paired-end reads remained for VL1 and VL2, respectively. These reads were mapped to the de novo assemblies using TopHat v2.0.9 [68], assuming an intron size of 5 to 5000 bp and otherwise default settings. The mapped reads were assembled into transcripts using Cufflinks v2.1.1 [69]. The predicted gene models could be partitioned into syntenic blocks with *V. dahliae* and/or *V. albo-atrum* using MCScanX, and default settings.

### Gene annotation

Genomes were annotated using the MAKER v2.30 pipeline [70]. First, genes were annotated based on similarity to the assembled transcripts of *V. dahliae* and *V. albo-atrum* using the est2genome option. This set of genes was used to train ab-initio predictors Augustus v2.5.5 [71], Genemark-ES v2.3 [72], and SNAP [73]. In addition to ab-initio predictors and protein sequences from *V. dahliae* and *V. albo-atrum*, the assembled transcript alignments were used as evidence for the prediction. This process was iterated two times with ab-initio retraining after each prediction. Functional annotation was generated using the automated pipelines Interproscan v5.44 [74] and Blast2GO v2.5 [75] using default settings. KOG terms were assessed using the online KOG classification tool (http:// weizhonglab.ucsd.edu/metagenomic-analysis).

### Repetitive sequences and transposable elements

Repeat libraries were constructed de novo using RepeatModeler v1.0.7 (http://www.repeatmasker.org/RepeatModeler.html). The repeat library was used to mask the entire genome using RepeatMasker version open-3.0.8, with Cross_Match version 0.990329, RepBase Update 9.04 RM database version 20,040,702. For calculation of percentage of genome masked by each repeat class, the ungapped genome size was used.

### Secretome and candidate effectors

Subcellular localization for all *V. longisporum* VL1 and VL2 proteins were predicted using WoLF PSORT v0.2 software [76]. The resulting putative extracellular group of proteins was further screened for the presence of signal peptides and signal peptide cleavage sites using the SignalP v4.0 program [77]. Subsequently, all proteins with signal peptides were analyzed for the presence of transmembrane domains using Phobius [78] and TMHMM version 2.0 [79]. The set of proteins with putative transmembrane domains was removed from the dataset. Remaining secreted proteins were then clustered in multiple enzymatic categories, dictated by the carbohydrate-active enzyme database, or CAZy [80]. Further divisions were made based on specific enzymatic groups (non-carbohydrates such as phosphatases and proteases), carbohydrate-binding capacity, and the remaining proteins were depicted as unknown. Short (<400 amino acids in mature chain) cysteine-rich (>4% cysteine content in mature chain) secreted proteins were denoted as potential effectors.

Clustering of secreted proteins and potential effector proteins was assessed as follows. First the pairwise distances between all secreted proteins was calculated and binned in 5 kb interval. The proportion of distances in each interval was then compared to a null-distribution obtained by randomly sample X proteins from the total set of proteins, where X is the number of secreted proteins, and likewise calculate the proportion of distances in each 5 kb interval. The random sampling was repeated 1000 times and the 95% confidence interval in proportion of pairwise distances in each 5 kb interval calculated. The process was repeated for the potential effectors.

### PCR and DNA sequencing

Fifty *nanograms* genomic DNA from both *V. longisporum* fungal isolates (VL-1 and VL-2) was used as a template and the Phusion high fidelity DNA polymerase (Thermo Fisher) was used in the following conditions for *MAT1–1-1* and *MAT1–2-1* gene amplifications: initial denaturation at 98 °C for 30 s followed by 35 cycles of 98 °C for 10 s, 58 °C for 30 s, 72 °C for 1 min, followed by final extension of 72 °C for 10 min before storage. Following primer sequences were used; *MAT1–1-1* For: 5′- ATG GAC GGT GTC CGA CCT GAAC-3′ and *MAT1–1-1*Rev: 5′ – TCA AAA GTA TGA AGC GAA CTG AGG GTGG-3′ and *MAT1–2-1*For: 5′- ATG TAT TTG TGT TCG TTA CAG ATC ACA TTTG-3′ and *MAT1–2-1* Rev.: 5’-CTA CAT GCT GGC CAA GAT GGC -3′. PCR products were purified from agarose gel using the GeneJET Gel Extraction Kit (Thermo Fisher), ligated to the pJET1.2/blunt vector using and cloned to *E. coli* DH5α cells (Thermo Fisher). Positive colonies were confirmed by restriction analysis and sent for Sanger sequencing (Macrogen Inc).

### Phylogenetic analysis

Maximum likelihood analysis was performed using RaXML v8.9.2, GTR+Γ or LG+Γ when appropriate [62], with random starting tree and rapid bootstrapping with 1000 or 10,000 replicates. Information on proteins used for the mating gene phylogeny, see Additional file 14.

## Additional files

Additional file 1: Top: Distribution of coverage when mapping *V. longisporum* reads to the *V. dahliae* reference genome of strain JR2 [22]. (PDF 109 kb)

Additional file 2: Distribution of fourfold degenerate transversion (4DTv)-rate between homologous genes in: *V. longisporum*; *V. longisporum* and *V. dahliae*; *V. longisporum* and *V. albo-atrum*; *V. dahliae* and *V. albo-atrum*. (PDF 156 kb)

Additional file 3: Maximum likelihood phylogenetic tree using a concatenation of 3592 genes. (PDF 92 kb)

Additional file 4: Maximum likelihood phylogenetic trees (RAXML, model GTR+Γ) for the: **A**. actin (*ACT*) gene, **B**. the elongation factor 1-alpha (*EF*) gene, **C**. the glyceraldehyde-3-phosphate dehydrogenase (*GP*) gene, **D**. the ribosomal internal transcribed spacer (ITS) region, **E**. the oxaloacetate transport (*OX*) gene, and **F**. the tryptophan synthase (*TS*) gene, using *Verticillium spp.* samples from Inderbitzin et al. [16] and homologous regions in the *V. longisporum* strain VL1 and VL2. (PDF 176 kb)

Additional file 5 Classes of transposable elements. (PDF 62 kb)

Additional file 6 Sequence homology and Pfam domain predictions for genes illustrated in Fig. 1. (XLSX 48 kb)

Additional file 7 Predicted secreted proteins. (PDF 54 kb)

Additional file 8 Predicted secreted proteins, arranged by KOG analysis. (PDF 64 kb)

Additional file 9 Candidate effectors (<400 aa) with cysteine rich residues in VL1. (PDF 75 kb)

Additional file 10 Candidate effectors (<400 aa) with cysteine rich residues in VL2. (PDF 76 kb)

Additional file 11 Cysteine content of all predicted secreted proteins plotted against sequence length (amino acids) and CAZy families and domains. (PDF 409 kb)

Additional file 12 Proportion of pairwise distances between genes in *V. longisporum* VL1 and VL2 genomes binned in 5 kb intervals. (PDF 78 kb)

Additional file 13 Distribution of predicted CAZy families. (PDF 64 kb)

Additional file 14 Accession numbers for proteins used for mating gene phylogeny in Fig. 2. (PDF 112 kb)

## Abbreviations

AA: Auxiliary activities
AFLP: Amplified fragment length polymorphism
CAZy: Carbohydrate active enzymes
CBM: Carbohydrate binding modules
CE: Carbohydrate esterase
CEGMA: Core eukaryotic gene mapping approach
GH: Glycoside hydrolase
GO: gene ontology
GT: glycosyl transferase
HMG: high mobility groups
KOG: eukaryotic orthologous groups
LINE: long interspersed nuclear element
LTR: long terminal repeat
MAT: mating type
NLP: necrosis and ethylene-inducing-like protein
PFAM: protein family database
RFLP: restriction length fragment polymorphism
SNP: single nucleotide polymorphism
TE: transposable elements
