# Supplementary material for: Analysis of the hybrid genomes of two field isolates of the soil-borne fungal species Verticillium longisporum
Source: BMC Genomics. 2018 Jan 3;19:14. doi: 10.1186/s12864-017-4407-x (PMC5753508; doi:10.1186/s12864-017-4407-x)
Supplement: Supplementary file 1 — Top: Distribution of coverage when mapping V. longisporum reads to the V. dahliae reference genome of strain JR2 [22]. (PDF 109 kb) [file 12864_2017_4407_MOESM1_ESM.pdf]

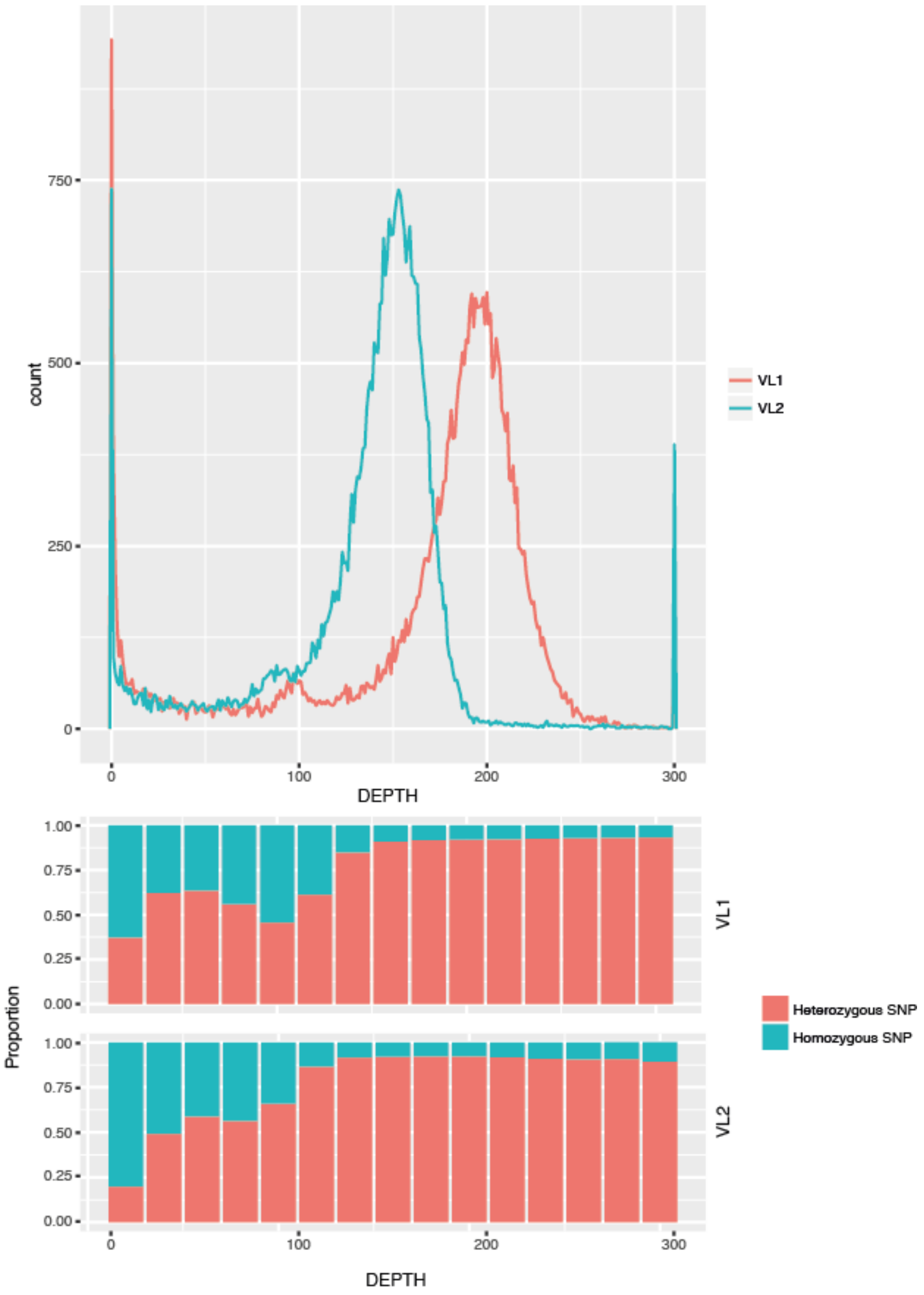


**Additional file 1:** Top: Distribution of coverage when mapping *V. longisporum* reads to the *V. dahliae* reference genome of strain JR2 [22]. Bottom: Heterozygosity of SNPs at different coverage in VL1 and VL2 genomes.
