# Supplementary material for: Analysis of the hybrid genomes of two field isolates of the soil-borne fungal species Verticillium longisporum
Source: BMC Genomics. 2018 Jan 3;19:14. doi: 10.1186/s12864-017-4407-x (PMC5753508; doi:10.1186/s12864-017-4407-x)
Supplement: Supplementary file 2 — Distribution of fourfold degenerate transversion (4DTv)-rate between homologous genes in: V. longisporum; V. longisporum and V. dahliae; V. longisporum and V. albo-atrum; V. dahliae and V. albo-atrum. (PDF 156 kb) [file 12864_2017_4407_MOESM2_ESM.pdf]

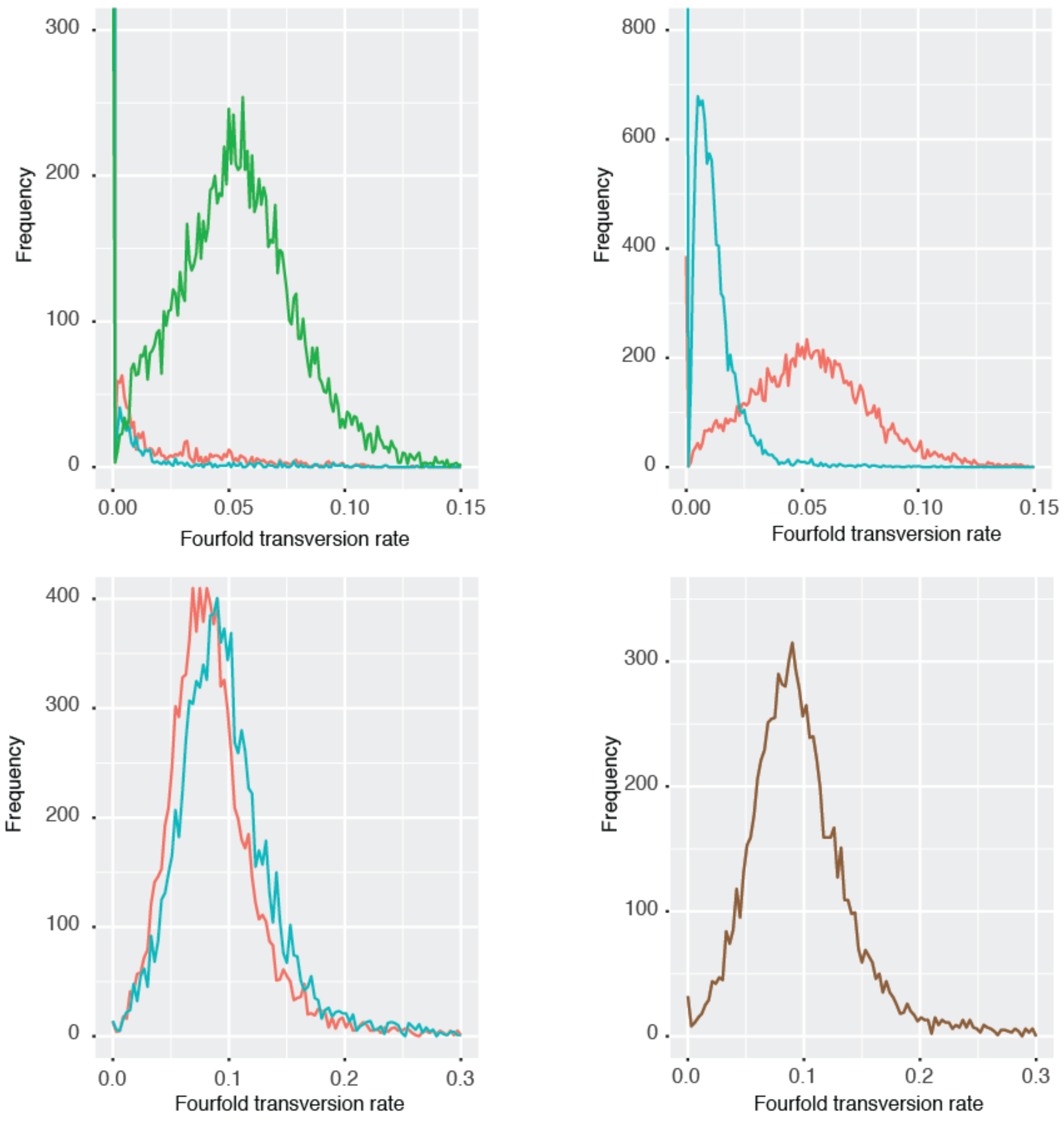

Additional file 2: Distribution of fourfold degenerate transversion (4DTv)-rate between homologous genes in: *V. longisporum* (top left); *V. longisporum* and *V. dahliae* (top right); *V. longisporum* and *V. albo-atrum* (bottom left); *V. dahliae* and *V. albo-atrum* (bottom right). Line color indicates comparison to the parental A genome (red), D genome (blue), between A and D genome (green) and to the joint set of A and D genome (brown). The top graphs have truncated y-scale as the frequency of 4DTv=0 was very high.
