# Supplementary material for: Analysis of the hybrid genomes of two field isolates of the soil-borne fungal species Verticillium longisporum
Source: BMC Genomics. 2018 Jan 3;19:14. doi: 10.1186/s12864-017-4407-x (PMC5753508; doi:10.1186/s12864-017-4407-x)
Supplement: Supplementary file 3 — Maximum likelihood phylogenetic tree using a concatenation of 3592 genes. (PDF 92 kb) [file 12864_2017_4407_MOESM3_ESM.pdf]

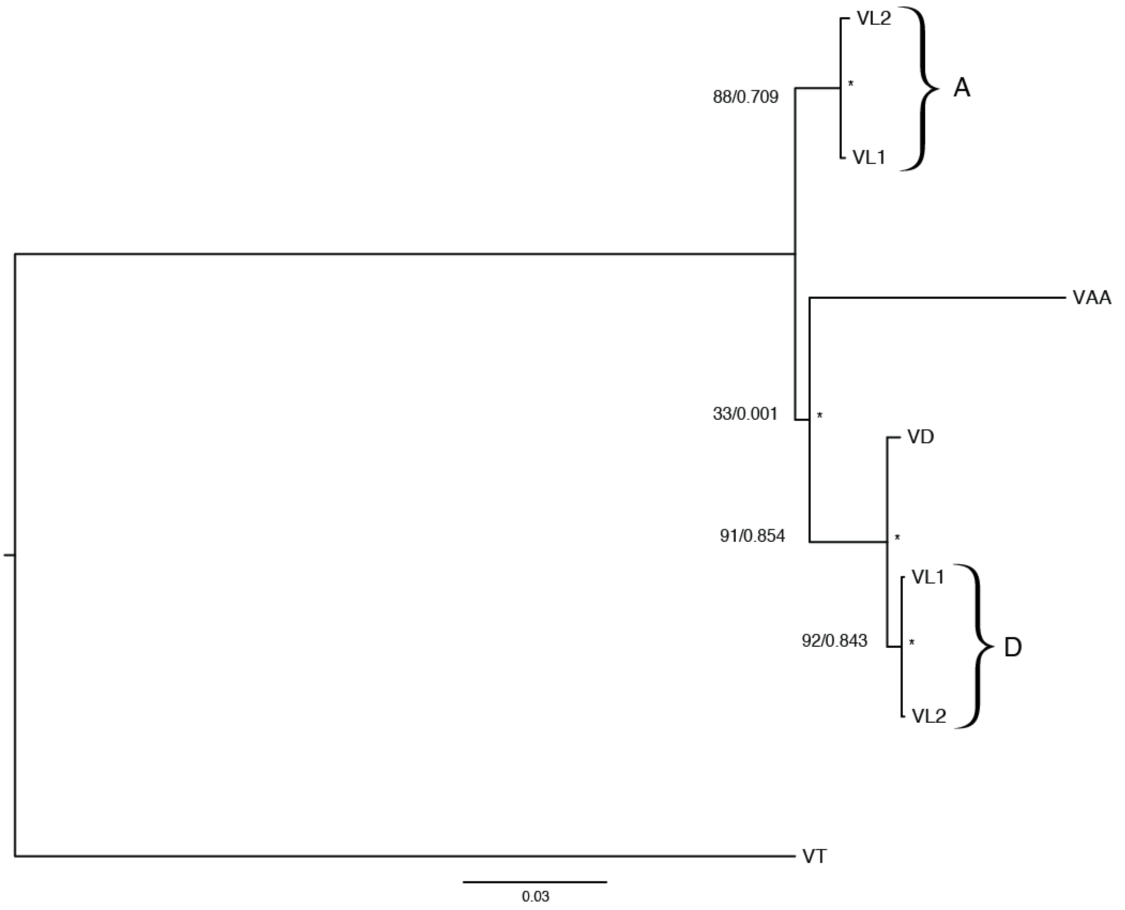

Additional file 3: Maximum likelihood phylogenetic tree using a concatenation of 3,592 genes. Asterisk indicates a bootstrap support value of 100 (1,000 replicates). For each internode the gene support value (left) and the internodal certainty value (right) are given. VL1 and VL2 represent the two *Verticillum longisporum* genomes, VD the *V. dahliae* reference genome, VAA, the *V. albo-atrum* genome. *V. tricorpus* (VT) is used as outgroup. Unit indicates number of amino acid changes per position.
