# Supplementary material for: Analysis of the hybrid genomes of two field isolates of the soil-borne fungal species Verticillium longisporum
Source: BMC Genomics. 2018 Jan 3;19:14. doi: 10.1186/s12864-017-4407-x (PMC5753508; doi:10.1186/s12864-017-4407-x)
Supplement: Supplementary file 4 — Maximum likelihood phylogenetic trees (RAXML, model GTR+Γ) for the: A. actin (ACT) gene, B. the elongation factor 1-alpha (EF) gene, C. the glyceraldehyde-3-phosphate dehydrogenase (GP) gene, D. the ribosomal internal transcribed spacer (ITS) region, E. the oxaloacetate transport (OX) gene, and F. the tryptophan synthase (TS) gene, using Verticillium spp. samples from Inderbitzin et al. [16] and homologous regions in the V. longisporum strain VL1 and VL2. (PDF 176 kb) [file 12864_2017_4407_MOESM4_ESM.pdf]

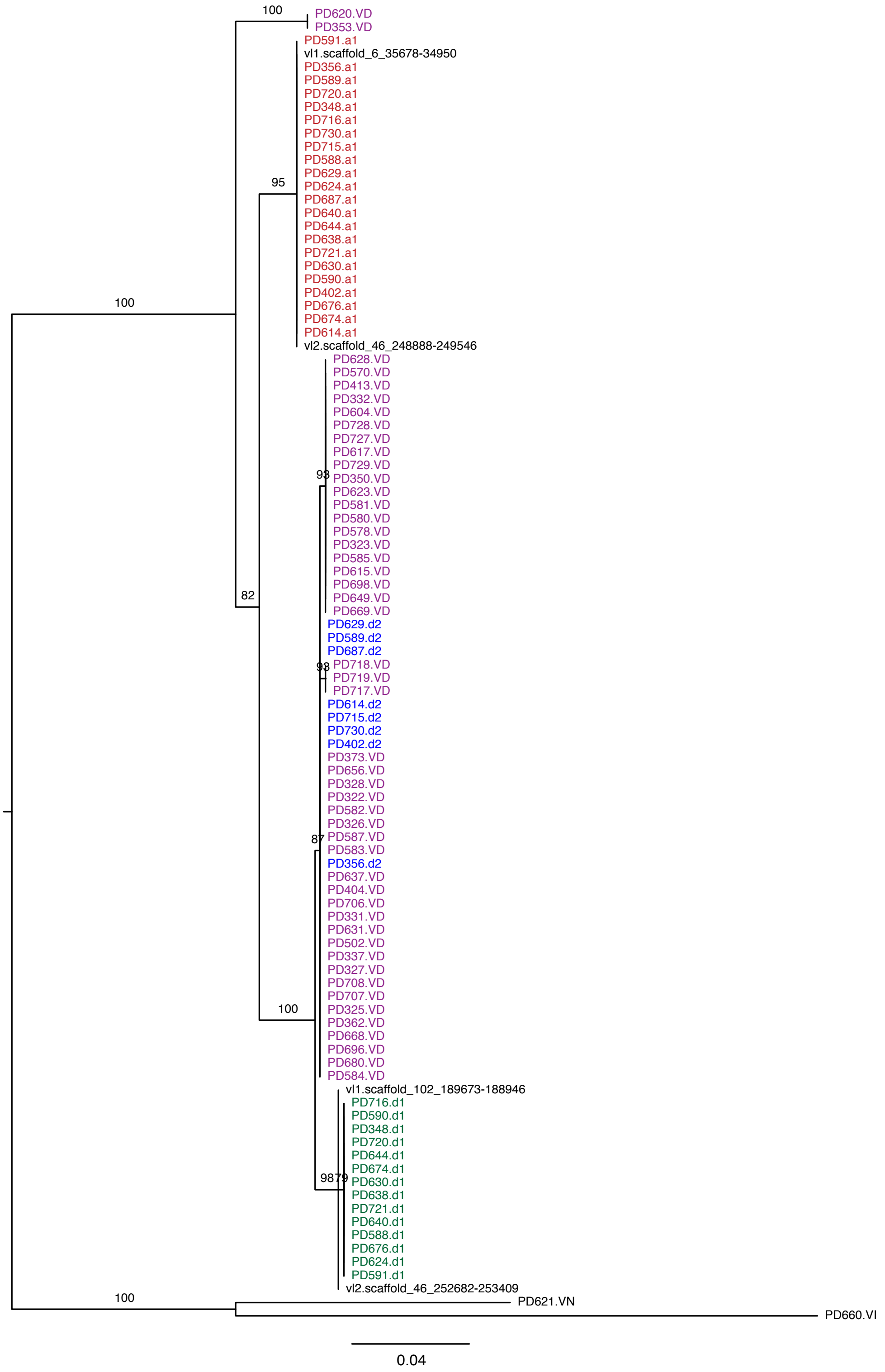

Additional file 4A: Maximum likelihood phylogenetic tree (RAXML, model GTR+Γ) for the actin (*ACT*) gene, using *Verticillium spp.* samples from Inderbitzin [16] (accession numbers HQ206921 - HQ207015) and homologous regions in *V. longisporum* strain VL1 and VL2. Nodes with weak bootstrap support (less than 50%) are collapsed. Unit indicates number of amino acid changes per position. Color code: red: *V. longisporum* lineage A1; lilac: *V. dahliae*; green: *V. longisporum* lineage D1; blue: *V. longisporum* lineage D2; black: samples sequenced in this study.

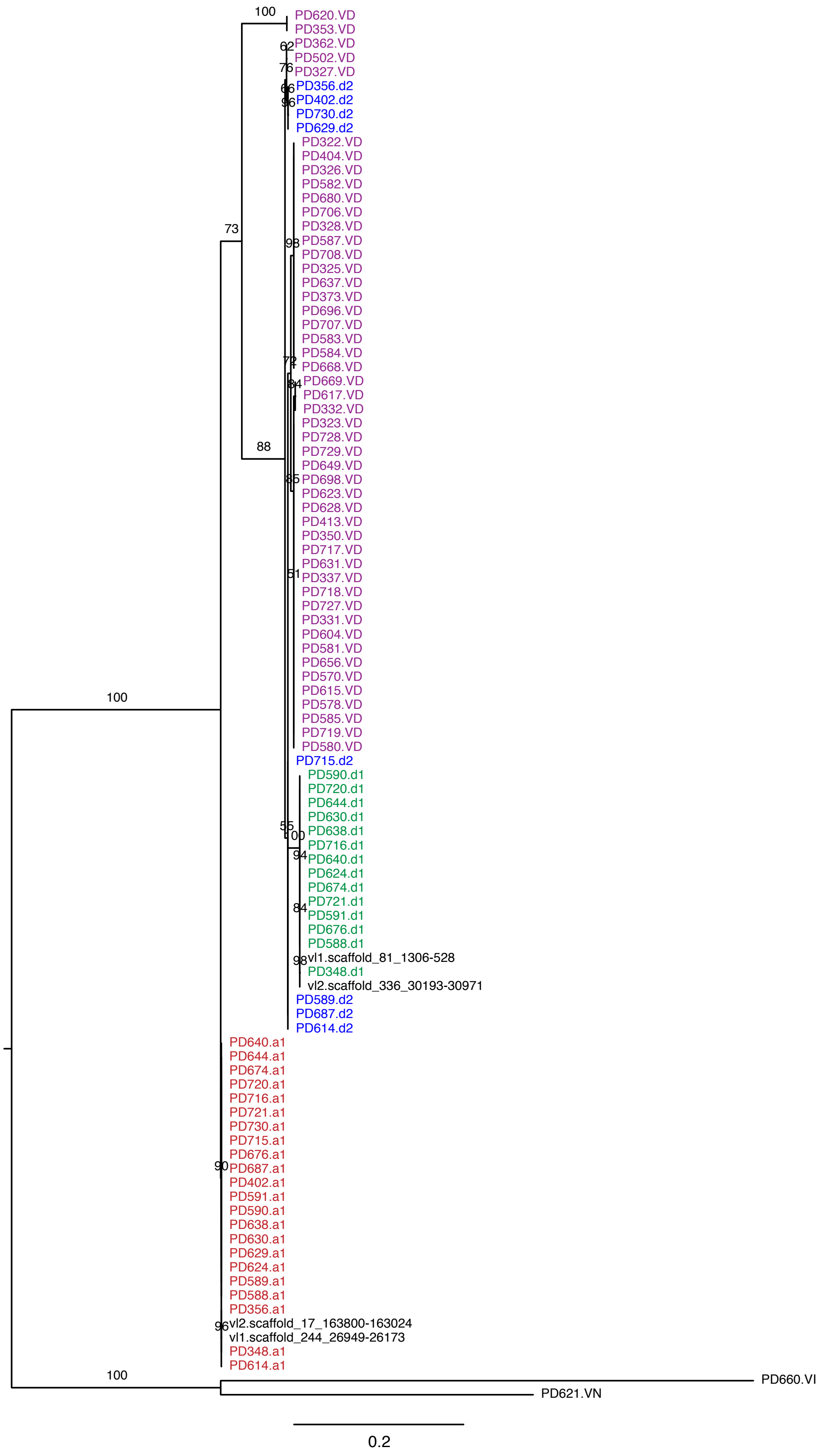

Additional file 4B: Maximum likelihood phylogenetic tree (RAXML, model GTR+Γ) for the elongation factor 1-alpha (*EF*) gene, using *Verticillium spp.* samples from Inderbitzin [16] (accession numbers HQ414624 - HQ414718) and homologous regions in *V. longisporum* strain VL1 and VL2. Unit indicates number of amino acid changes per position. Color code: red: *V. longisporum* lineage A1; lilac: *V. dahliae*; green: *V. longisporum* lineage D1; blue: *V. longisporum* lineage D2; black: samples sequenced in this study.

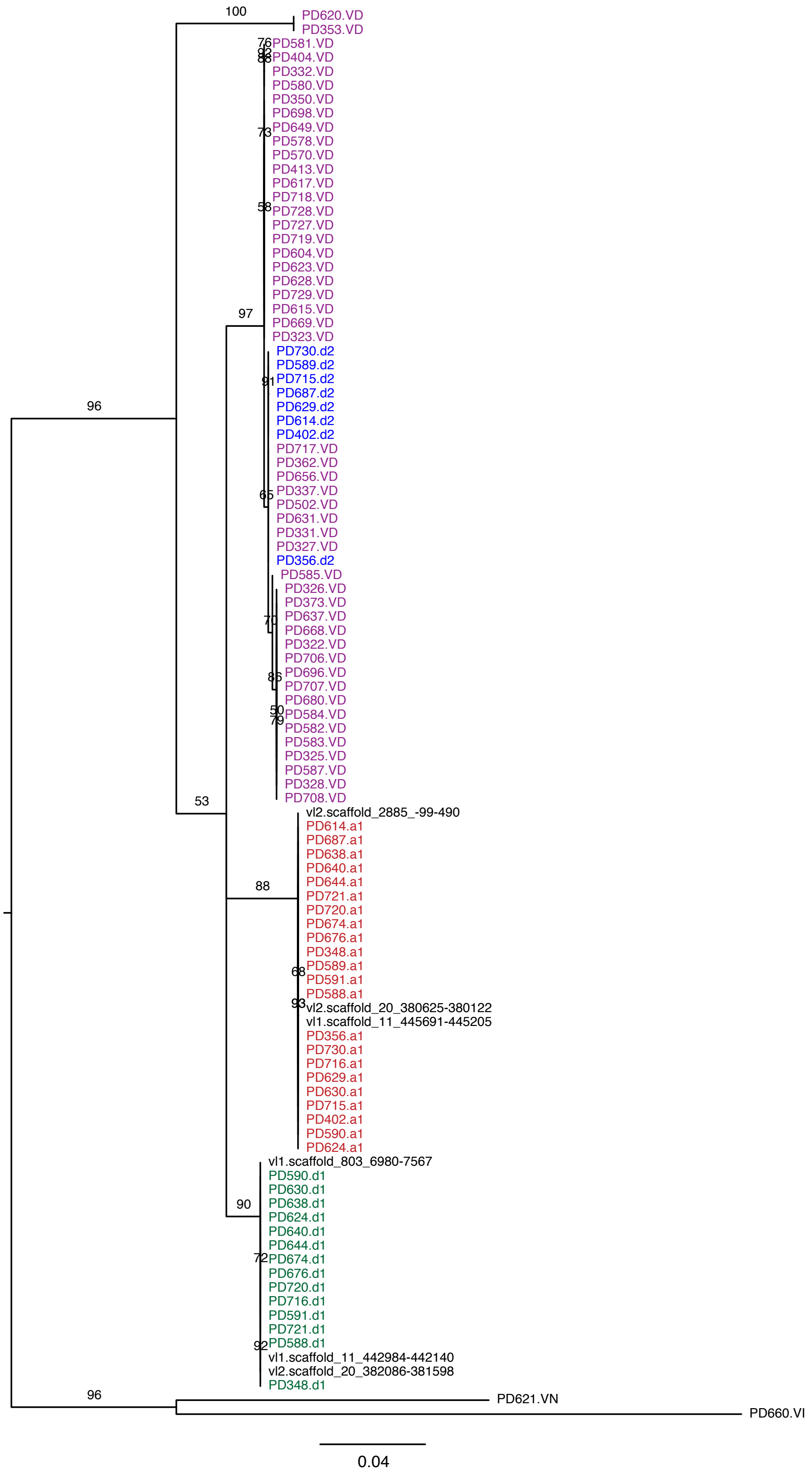

Additional file 4C: Maximum likelihood phylogenetic tree (RAXML, model GTR+Γ) for the glyceraldehyde-3-phosphate (*GP*) dehydrogenase gene, using *Verticillium spp.* samples from Inderbitzin [16] (accession numbers HQ414719 - HQ414813) and homologous regions in *V. longisporum* strain VL1 and VL2. Nodes with weak bootstrap support (less than 50%) are collapsed. Unit indicates number of amino acid changes per position. Color code: red: *V. longisporum* lineage A1; lilac: *V. dahliae*; green: *V. longisporum* lineage D1; blue: *V. longisporum* lineage D2; black: samples sequenced in this study.

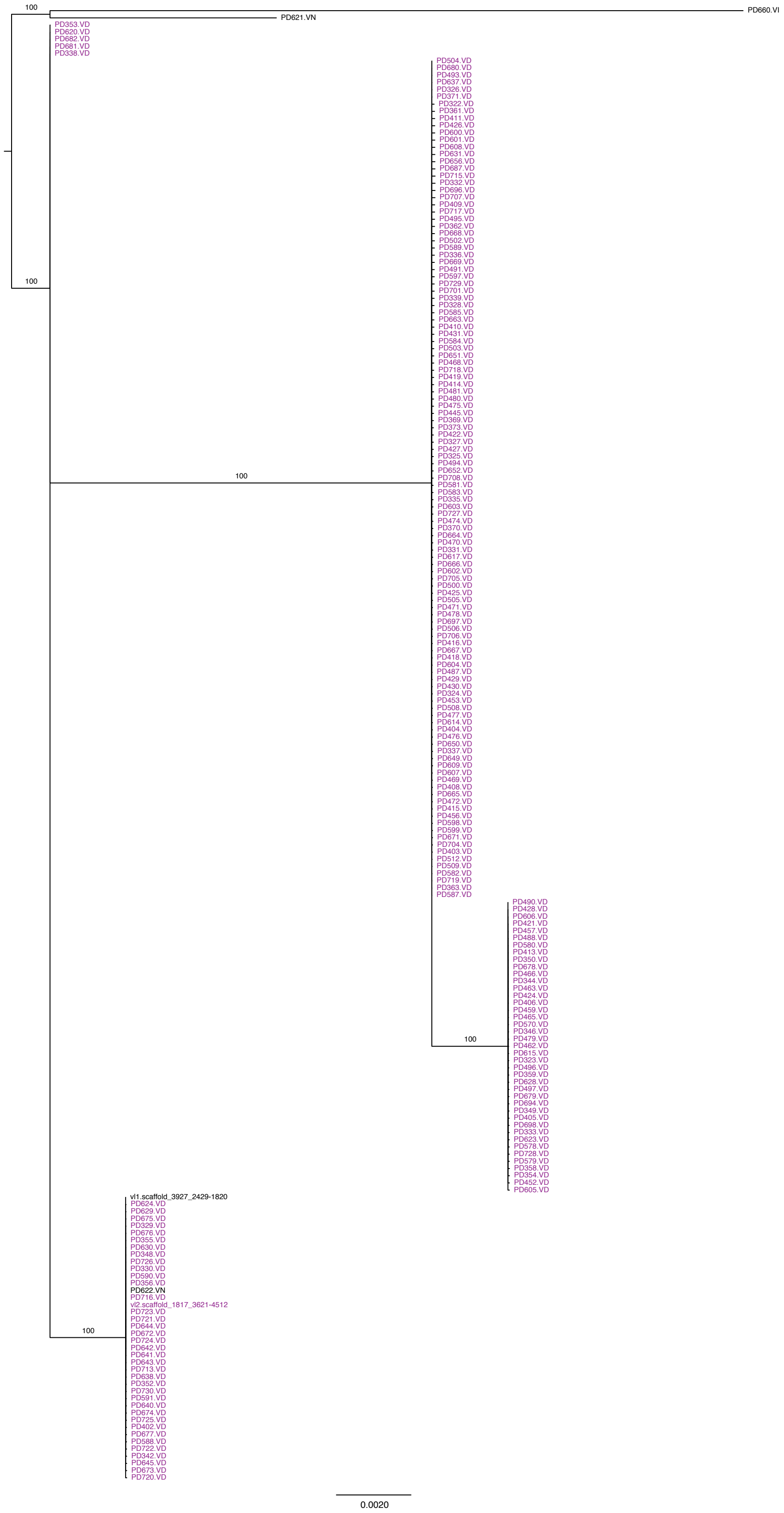

Additional file 4D: Maximum likelihood phylogenetic tree (RAXML, model GTR+Γ) for the ribosomal internal transcribed spacer (ITS) region, using *Verticillium spp.* samples from Inderbitzin [16] (accession numbers HQ206718 - HQ206920) and homologous regions in *V. longisporum* strain VL1 and VL2. Nodes with weak bootstrap support (less than 50%) are collapsed. Unit indicates number of amino acid changes per position. Color code: red: *V. longisporum* lineage A1; lilac: *V. dahliae*; green: *V. longisporum* lineage D1; blue: *V. longisporum* lineage D2; black: samples sequenced in this study.

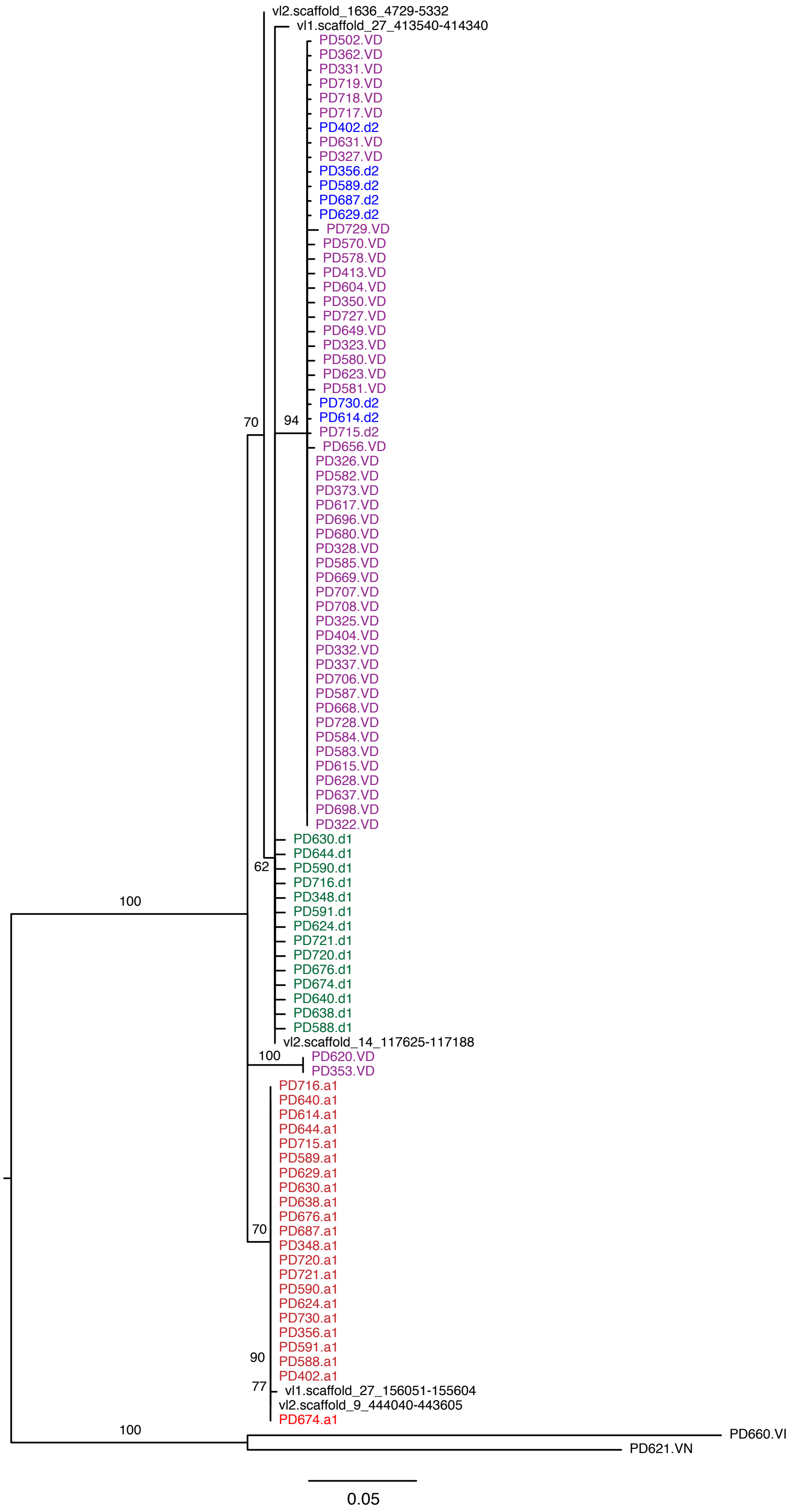

Additional file 4E. Maximum likelihood phylogenetic tree (RAXML, model GTR+Γ) for the oxaloacetate transport (*OX*) gene, using *Verticillium spp.* samples from Inderbitzin [16] (accession numbers HQ414814 - HQ414908) and homologous regions in *V. longisporum* strain VL1 and VL2. Nodes with weak bootstrap support (less than 50%) are collapsed. Unit indicates number of amino acid changes per position. Color code: red: *V. longisporum* lineage A1; lilac: *V. dahliae*; green: *V. longisporum* lineage D1; blue: *V. longisporum* lineage D2; black: samples sequenced in this study.

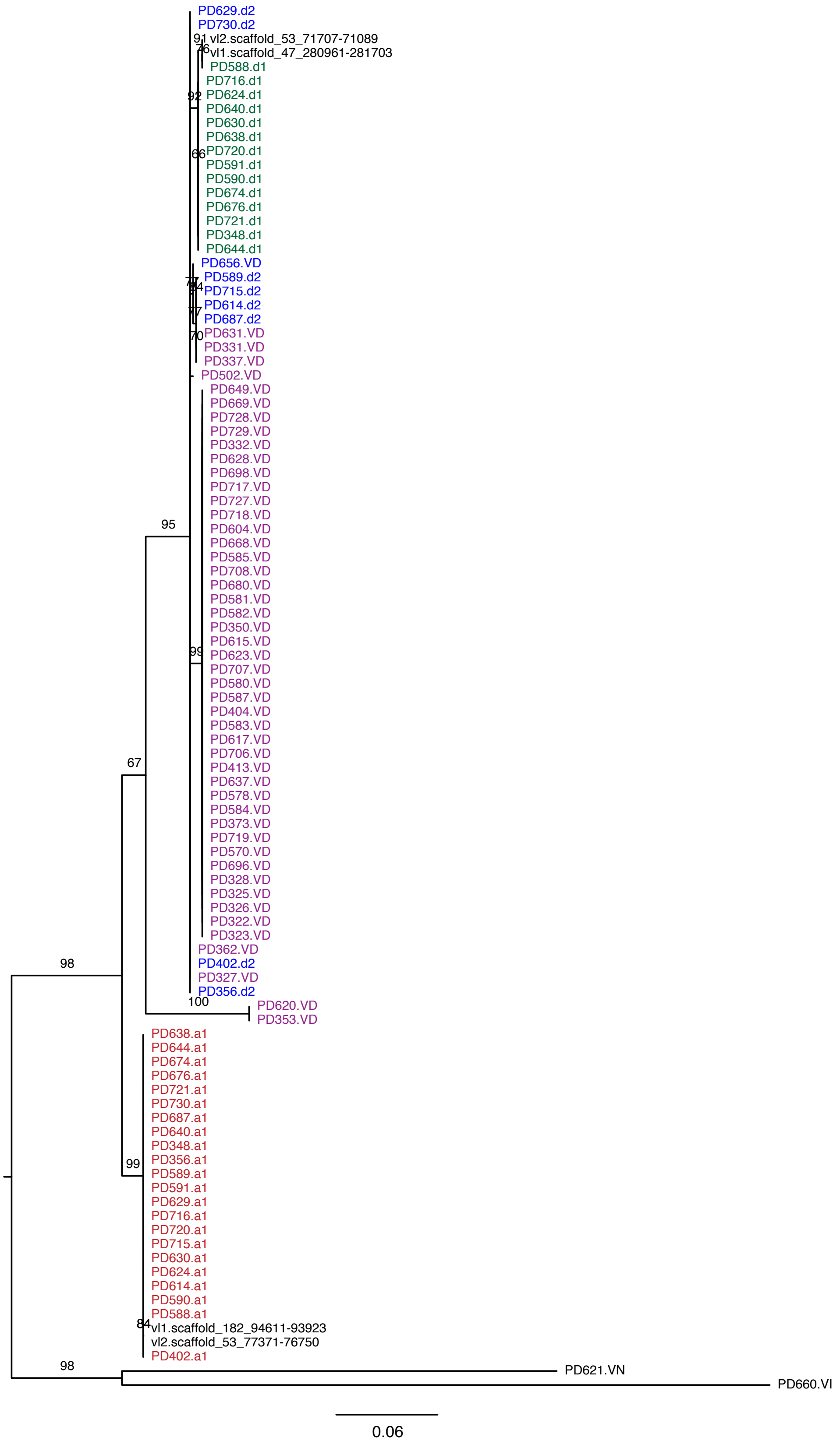

Additional file 4F: Maximum likelihood phylogenetic tree (RAXML, model GTR+Γ) for the tryptophan synthase (*TS*) gene, using *Verticillium spp.* samples from Inderbitzin [16] (accession numbers HQ414909 - HQ415003) and homologous regions in *V. longisporum* strain VL1 and VL2. Nodes with weak bootstrap support (less than 50%) are collapsed. Unit indicates number of amino acid changes per position. Color code: red: *V. longisporum* lineage A1; lilac: *V. dahliae*; green: *V. longisporum* lineage D1; blue: *V. longisporum* lineage D2; black: samples sequenced in this study.
