# Supplementary material for: Analysis of the hybrid genomes of two field isolates of the soil-borne fungal species Verticillium longisporum
Source: BMC Genomics. 2018 Jan 3;19:14. doi: 10.1186/s12864-017-4407-x (PMC5753508; doi:10.1186/s12864-017-4407-x)
Supplement: Supplementary file 5 — Classes of transposable elements. (PDF 62 kb) [file 12864_2017_4407_MOESM5_ESM.pdf]

Additional file 5: Classes of transposable elements identified in the genomes of *V. longisporum* VL1 and VL2.

Class	Family	Amount VL1	Percentage (%)	Amount VL 2	Percentage (%)
I	LINE/1	0	0	52	0.08
	LINE/Tad1	82	0.04	27	0.03
	LINE/L1	45	0.06	0	0
	LTR/Copia	1160	0.42	3222	0.96
	LTR/ERVK	32	0.05	46	0.04
	LTR/Gypsy	1338	0.49	4402	1.64
	LTR/Pao	114	0.07	0	0
	SINE	102	0.02	70	0.01
II	DNA/Crypton	31	0.08	0	0
	DNA/PIF-Harbinger	0	0	33	0.03
	DNA/TcMar-Fot1	251	0.12	187	0.10
	DNA/Tc-Mar-Pogo	0	0	86	0.06
	DNA/hAT-Ac	0	0	127	0.09
Total			1.35		3.04
Unclassified		10,009	3.13	29,895	7.87

LTR: long terminal repeat retrotransposon, LINE: long interspersed elements, SINE: short interspersed elements
