# Supplementary material for: Analysis of the hybrid genomes of two field isolates of the soil-borne fungal species Verticillium longisporum
Source: BMC Genomics. 2018 Jan 3;19:14. doi: 10.1186/s12864-017-4407-x (PMC5753508; doi:10.1186/s12864-017-4407-x)
Supplement: Supplementary file 7 — Predicted secreted proteins. (PDF 54 kb) [file 12864_2017_4407_MOESM7_ESM.pdf]

Additional file 7: Predicted secreted proteins in *V. longisporum* (VL1 and VL2), *V. dahliae* VdLs.17 (VD) and *V. albo-atrum* VaMs.102 (VAA).

	**VL1**	**VL2**	**VD**[20]	**VAA**[20]
Total secreted	1,281	1,251	746	767
Cysteine-rich proteins	204	203	127	124
LysM	11	8	7	6
NLP	12	15	8	7

NLP: necrosis and ethylene-inducing-like protein.
