# Supplementary material for: Analysis of the hybrid genomes of two field isolates of the soil-borne fungal species Verticillium longisporum
Source: BMC Genomics. 2018 Jan 3;19:14. doi: 10.1186/s12864-017-4407-x (PMC5753508; doi:10.1186/s12864-017-4407-x)
Supplement: Supplementary file 8 — Predicted secreted proteins, arranged by KOG analysis. (PDF 64 kb) [file 12864_2017_4407_MOESM8_ESM.pdf]

Additional file 8: Predicted secreted proteins, arranged by KOG analysis.

	**VL1**	**VL2**	**VD[20]**	**VAA[20]**
Total	1281	1251	746	767
With KOG	488	484	258	268
M	20	18	9	10
N	0	0	0	0
O	110	110	54	58
T	65	58	30	27
U	29	26	12	14
W	9	9	6	6
V	2	3	3	3
Y	4	3	1	2
Z	45	35	21	11
A	11	5	4	9
B	9	4	3	2
J	24	16	4	1
K	37	38	17	14
L	6	12	3	5
C	38	34	25	25
D	14	15	6	4
E	27	25	9	10
F	7	9	4	3
G	62	58	25	30
H	2	2	1	0
I	17	21	10	12
P	21	19	13	12
Q	24	27	14	16
R	83	81	51	47
S	32	31	19	13

A: RNA processing and modification, B: chromatin structure and dynamics, C: energy production and conversion, D: cell cycle control, cell division and chromosome partitioning, E: amino acid transport and metabolism, F: nucleotide transport and metabolism, G: carbohydrate transport and metabolism, H: coenzyme transport and metabolism, I: lipid transport and metabolism, J: translation, ribosomal structure and biogenesis, K: transcription, L: replication, recombination and repair, M: cell wall, membrane or envelope biogenesis, N: cell motility, O: post-translational modification, protein turnover chaperones, P: inorganic ion transport and metabolism, Q: secondary metabolites biosynthesis transport and catabolism, R: general function prediction only, S: function unknown, T: signal transduction, U: intracellular trafficking secretion and vesicular transport, V: defense mechanisms, W: extracellular structures, Y: nuclear structure, Z: cytoskeleton.
