# Supplementary material for: Analysis of the hybrid genomes of two field isolates of the soil-borne fungal species Verticillium longisporum
Source: BMC Genomics. 2018 Jan 3;19:14. doi: 10.1186/s12864-017-4407-x (PMC5753508; doi:10.1186/s12864-017-4407-x)
Supplement: Supplementary file 9 — Candidate effectors (<400 aa) with cysteine rich residues in VL1. (PDF 75 kb) [file 12864_2017_4407_MOESM9_ESM.pdf]

Additional file 9: Candidate effectors (<400 aa) with cysteine rich residues in the *V. longisporum* VL1 secretome.

ID	PFAM	CAZy
VL1_T00002192_1	Cerato-platanin	NA
VL1_T00002194_1	Cerato-platanin	NA
VL1_T00000007_1	CFEM domain	NA
VL1_T00000785_1	CFEM domain	NA
VL1_T00001144_1	CFEM domain	NA
VL1_T00001159_1	CFEM domain	NA
VL1_T00002272_1	CFEM domain	NA
VL1_T00005073_1	CFEM domain	NA
VL1_T00005074_1	CFEM domain	NA
VL1_T00007470_1	CFEM domain	NA
VL1_T00008069_1	CFEM domain	NA
VL1_T00008775_1	CFEM domain	NA
VL1_T00009247_1	CFEM domain	NA
VL1_T00009674_1	CFEM domain	NA
VL1_T00013237_1	CFEM domain	NA
VL1_T00013238_1	CFEM domain	NA
VL1_T00014125_1	Chitin binding domain	NA
VL1_T00018452_1	Chitin binding domain	AA10
VL1_T00003943_1	Chitin binding domain;Starch binding domain	AA10; CBM20
VL1_T00012400_1	Chitin recognition protein	CBM18
VL1_T00012066_1	Common central domain of tyrosinase	NA
VL1_T00013797_1	Common central domain of tyrosinase	NA
VL1_T00008342_1	Cutinase	CE5
VL1_T00004714_1	Cytidine and deoxycytidylate deaminase zinc-binding region	NA
VL1_T00004899_1	FAD binding domain	NA
VL1_T00000689_1	Fungal cellulose binding domain	CE1; CBM1
VL1_T00007473_1	Fungal cellulose binding domain	CBM1
VL1_T00009249_1	Fungal cellulose binding domain	CBM1
VL1_T00007399_1	Fungal cellulose binding domain;Glycosyl hydrolase family 12	GH12; CBM1
VL1_T00005957_1	Fungal cellulose binding domain;Glycosyl hydrolase family 45	GH45; CBM1
VL1_T00003773_1	Fungal cellulose binding domain;Glycosyl hydrolase family 61	AA9; CBM1
VL1_T00011228_1	Fungal cellulose binding domain;Glycosyl hydrolase family 61	AA9; CBM1
VL1_T00012191_1	Fungal cellulose binding domain;Glycosyl hydrolase family 61	AA9; CBM1
VL1_T00002125_1	Fungal hydrophobin	NA
VL1_T00004912_1	Fungal hydrophobin	NA
VL1_T00005496_1	Fungal hydrophobin	NA
VL1_T00005504_1	Fungal hydrophobin	NA
VL1_T00009136_1	Fungal hydrophobin	NA
VL1_T00011825_1	Fungal hydrophobin	NA
VL1_T00012793_1	Fungal hydrophobin	NA
VL1_T00014482_1	Fungal hydrophobin	NA
VL1_T00016215_1	Fungal hydrophobin	NA
VL1_T00010132_1	Glycosyl hydrolase catalytic core	GH128
VL1_T00013898_1	Glycosyl hydrolase family 12;Fungal cellulose binding domain	GH12; CBM1
VL1_T00007491_1	Glycosyl hydrolase family 45	GH45
VL1_T00010432_1	Glycosyl hydrolase family 45	GH45
VL1_T00017261_1	Glycosyl hydrolase family 45	GH45
VL1_T00006100_1	Glycosyl hydrolase family 61	AA9
VL1_T00014492_1	Glycosyl hydrolase family 7	GH7
VL1_T00017253_1	Glycosyl hydrolase family 7	GH7
VL1_T00007440_1	Glycosyl hydrolases family 28	GH28
VL1_T00016733_1	Glycosyl hydrolases family 28	GH28
VL1_T00008255_1	Lipocalin-like domain	NA
VL1_T00009637_1	Lipocalin-like domain	NA
VL1_T00000680_1	LysM domain	CBM50
VL1_T00007527_1	LysM domain	NA
VL1_T00001182_1	Pectate lyase	PL3
VL1_T00002179_1	Pectate lyase	PL3
VL1_T00003736_1	Pectate lyase	PL3
VL1_T00004802_1	Pectate lyase	PL3
VL1_T00005407_1	Pectate lyase	PL3
VL1_T00006187_1	Pectate lyase	PL3
VL1_T00009937_1	Pectate lyase	PL3
VL1_T00011753_1	Pectate lyase	PL3
VL1_T00016173_1	Pectate lyase	PL3
VL1_T00019388_1	Pectate lyase	PL3
VL1_T00003220_1	Peptidase inhibitor I78 family	NA
VL1_T00003720_1	Phage lysozyme	GH24
VL1_T00008348_1	Pregnancy-associated plasma protein-A	NA
VL1_T00009465_1	Pregnancy-associated plasma protein-A	NA
VL1_T00014035_1	Prokaryotic phospholipase A2	NA
VL1_T00001858_1	ribonuclease	NA

VL1_T00002070_1	Ribonuclease T2 family	NA
VL1_T00014637_1	Ribonuclease T2 family	NA
VL1_T00017709_1	RING-H2 zinc finger;Thioredoxin	NA
VL1_T00011857_1	S1/P1 Nuclease	NA
VL1_T00006681_1	Scytalone dehydratase;Fungal cellulose binding domain	CBM1
VL1_T00007730_1	SelR domain	NA
VL1_T00018435_1	Thaumatin family	NA
VL1_T00014329_1	Ubiquitin 3 binding protein But2 C-terminal domain	NA
VL1_T00009155_1	WSC domain	NA
