# Supplementary material for: Analysis of the hybrid genomes of two field isolates of the soil-borne fungal species Verticillium longisporum
Source: BMC Genomics. 2018 Jan 3;19:14. doi: 10.1186/s12864-017-4407-x (PMC5753508; doi:10.1186/s12864-017-4407-x)
Supplement: Supplementary file 10 — Candidate effectors (<400 aa) with cysteine rich residues in VL2. (PDF 76 kb) [file 12864_2017_4407_MOESM10_ESM.pdf]

**Additional file 10:** Candidate effectors (<400 aa) with cysteine rich residues in the *V. longisporum* VL2 secretome.

| ID | PFAM | CAZy |
|---|---|---|
| VL2_T00018694_1 | 4Fe-4S dicluster domain | NA |
| VL2_T00016971_1 | Asparaginase | NA |
| VL2_T00019950_1 | Beta-glucosidase (SUN family) | NA |
| VL2_T00003682_1 | Cerato-platanin | NA |
| VL2_T00000099_1 | CFEM domain | NA |
| VL2_T00000101_1 | CFEM domain | NA |
| VL2_T00002822_1 | CFEM domain | NA |
| VL2_T00002823_1 | CFEM domain | NA |
| VL2_T00004752_1 | CFEM domain | NA |
| VL2_T00006091_1 | CFEM domain | NA |
| VL2_T00006295_1 | CFEM domain | NA |
| VL2_T00006303_1 | CFEM domain | NA |
| VL2_T00010190_1 | CFEM domain | NA |
| VL2_T00010942_1 | CFEM domain | NA |
| VL2_T00012496_1 | CFEM domain | NA |
| VL2_T00012497_1 | CFEM domain | NA |
| VL2_T00014666_1 | CFEM domain | NA |
| VL2_T00014995_1 | CFEM domain | NA |
| VL2_T00016609_1 | Chitin binding domain | NA |
| VL2_T00006177_1 | Chitin binding domain;Starch binding domain | AA10; CBM20 |
| VL2_T00006181_1 | Chitin binding domain;Starch binding domain | AA10; CBM20 |
| VL2_T00009563_1 | Chitin recognition protein | CBM18 |
| VL2_T00012874_1 | Common central domain of tyrosinase | NA |
| VL2_T00015782_1 | Common central domain of tyrosinase | NA |
| VL2_T00000074_1 | CVNH domain | NA |
| VL2_T00014303_1 | Cytidine and deoxycytidylate deaminase zinc-binding region | NA |
| VL2_T00005826_1 | Deuterolysin metalloprotease (M35) family | NA |
| VL2_T00003718_1 | Domain of unknown function (DUF1929);PAN domain | NA |
| VL2_T00004333_1 | Fungal cellulose binding domain | CBM1 |
| VL2_T00008643_1 | Fungal cellulose binding domain | CBM1 |
| VL2_T00010192_1 | Fungal cellulose binding domain | CBM1 |
| VL2_T00014993_1 | Fungal cellulose binding domain | CBM1 |
| VL2_T00016065_1 | Fungal cellulose binding domain | CE1; CBM1 |
| VL2_T00019046_1 | Fungal cellulose binding domain | CBM1 |
| VL2_T00004116_1 | Fungal cellulose binding domain;Glycosyl hydrolase family 12 | GH12; CBM1 |
| VL2_T00005280_1 | Fungal cellulose binding domain;Glycosyl hydrolase family 7 | CBM1 |
| VL2_T00015626_1 | Fungal cellulose binding domain;Ribosomal protein S27a;Cellulase (glycosyl hydrolase family 5) | CBM1 |
| VL2_T00001840_1 | Fungal hydrophobin | NA |
| VL2_T00005460_1 | Fungal hydrophobin | NA |
| VL2_T00009849_1 | Fungal hydrophobin | NA |
| VL2_T00014958_1 | Fungal hydrophobin | NA |
| VL2_T00017477_1 | Fungal hydrophobin | NA |
| VL2_T00017311_1 | Fungal Zn(2)-Cys(6) binuclear cluster domain | NA |
| VL2_T00016380_1 | Glycosyl hydrolase catalytic core | GH128 |
| VL2_T00003044_1 | Glycosyl hydrolase family 12;Fungal cellulose binding domain | GH12; CBM1 |
| VL2_T00002117_1 | Glycosyl hydrolase family 45 | GH45 |
| VL2_T00006507_1 | Glycosyl hydrolase family 45 | GH45 |
| VL2_T00001512_1 | Glycosyl hydrolase family 61 | AA9 |
| VL2_T00003338_1 | Glycosyl hydrolase family 61 | AA9 |
| VL2_T00003342_1 | Glycosyl hydrolase family 61 | AA9 |
| VL2_T00002899_1 | Glycosyl hydrolase family 7 | GH7 |
| VL2_T00007845_1 | Glycosyl hydrolase family 7 | GH7 |
| VL2_T00015130_1 | Glycosyl hydrolase family 7 | GH7 |
| VL2_T00015727_1 | Glycosyl hydrolase family 7 | GH7 |
| VL2_T00010420_1 | Glycosyl hydrolases family 28 | GH28 |
| VL2_T00010426_1 | Glycosyl hydrolases family 28 | GH28 |
| VL2_T00010077_1 | Glycosyl hydrolases family 6;Glycosyl hydrolases family 6;Fungal cellulose binding domain | CBM1 |
| VL2_T00008547_1 | Imidazoleglycerol-phosphate dehydratase | NA |
| VL2_T00012193_1 | LysM domain | NA |
| VL2_T00015946_1 | LysM domain | CBM50 |
| VL2_T00007162_1 | Pectate lyase | PL3 |
| VL2_T00007493_1 | Pectate lyase | PL3 |
| VL2_T00007503_1 | Pectate lyase | PL3 |
| VL2_T00007515_1 | Pectate lyase | PL3 |
| VL2_T00009422_1 | Pectate lyase | PL3 |
| VL2_T00010011_1 | Pectate lyase | PL3 |
| VL2_T00014497_1 | Pectate lyase | PL3 |
| VL2_T00015588_1 | Pectate lyase | PL3 |
| VL2_T00019515_1 | Pectate lyase | PL3 |
| VL2_T00002847_1 | Peptidase inhibitor I78 family | NA |

| | | |
|---|---|---|
| VL2_T00007528_1 | Phage lysozyme | GH24 |
| VL2_T00009750_1 | Pregnancy-associated plasma protein-A | NA |
| VL2_T00016417_1 | Pregnancy-associated plasma protein-A | NA |
| VL2_T00014755_1 | Prokaryotic phospholipase A2 | NA |
| VL2_T00013642_1 | Ribonuclease T2 family | NA |
| VL2_T00009309_1 | Scytalone dehydratase;Fungal cellulose binding domain | CBM1 |
| VL2_T00009852_1 | Subtilase family | NA |
| VL2_T00000420_1 | Tannase and feruloyl esterase | NA |
| VL2_T00018361_1 | Thioredoxin;RING-H2 zinc finger | NA |
| VL2_T00006307_1 | Transcription factor S-II (TFIIS);Thaumatin family | NA |
| VL2_T00001021_1 | Vacuolar protein sorting-associated protein 26 | NA |
| VL2_T00002475_1 | WSC domain | NA |
| VL2_T00002480_1 | WSC domain | NA |
| VL2_T00003033_1 | WSC domain | NA |
