# Supplementary material for: Analysis of the hybrid genomes of two field isolates of the soil-borne fungal species Verticillium longisporum
Source: BMC Genomics. 2018 Jan 3;19:14. doi: 10.1186/s12864-017-4407-x (PMC5753508; doi:10.1186/s12864-017-4407-x)
Supplement: Supplementary file 11 — Cysteine content of all predicted secreted proteins plotted against sequence length (amino acids) and CAZy families and domains. (PDF 409 kb) [file 12864_2017_4407_MOESM11_ESM.pdf]

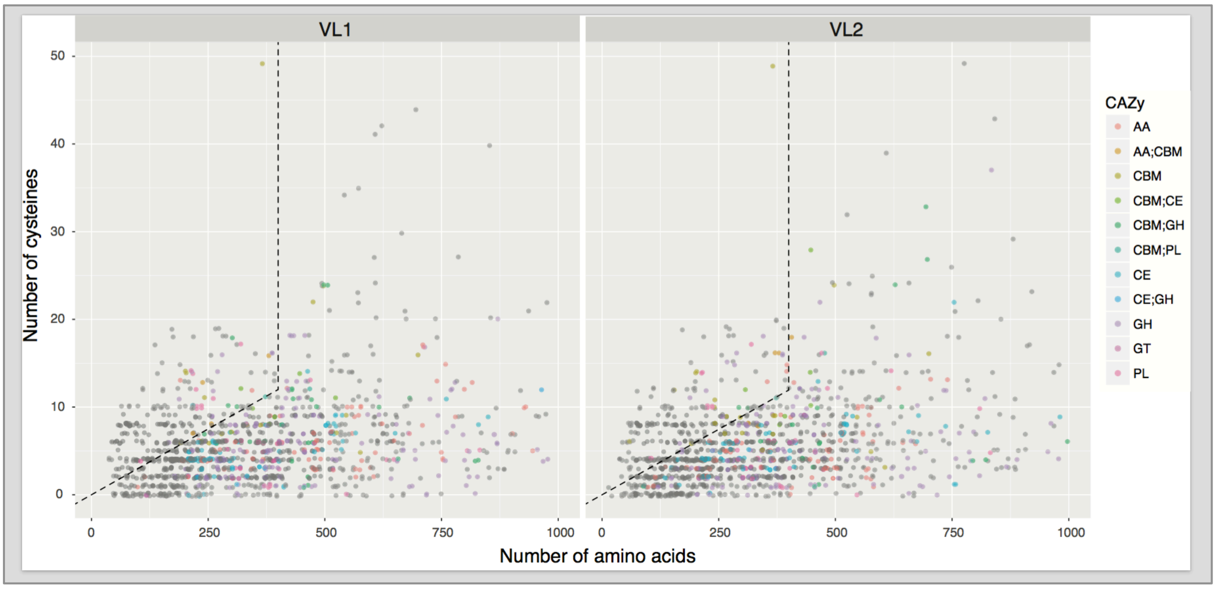


**Additional file 11:** Cysteine content of all secreted proteins predicted plotted against sequence length (amino acids) and CAZy families and domains. The dotted line indicates the group of cysteine riche, and small secreted proteins (<400 amino acids) to the left. CAZy-domains are indicted to the right.
