# Supplementary material for: Analysis of the hybrid genomes of two field isolates of the soil-borne fungal species Verticillium longisporum
Source: BMC Genomics. 2018 Jan 3;19:14. doi: 10.1186/s12864-017-4407-x (PMC5753508; doi:10.1186/s12864-017-4407-x)
Supplement: Supplementary file 12 — Proportion of pairwise distances between genes in V. longisporum VL1 and VL2 genomes binned in 5 kb intervals. (PDF 78 kb) [file 12864_2017_4407_MOESM12_ESM.pdf]

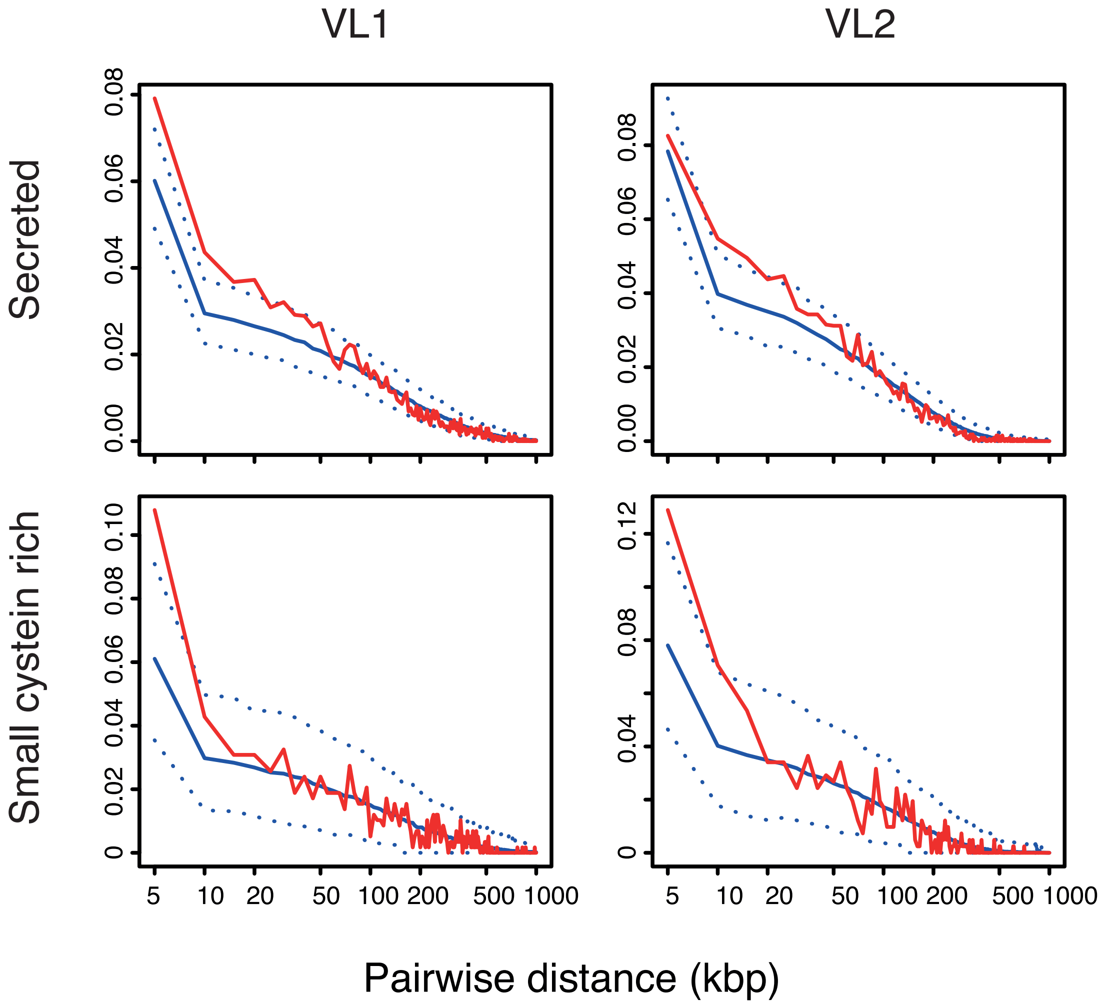


**Additional file 12:** Proportion of pairwise distances between genes in *V. longisporum* VL1 and VL2 genomes, binned in 5kb intervals, secreted proteins (top), candidate effectors (below). Solid red line: realized distribution, blue solid line: average and 95% CI (dotted) distribution in random selection of an equally sized pool of genes (1,000 permutations).
