# Supplementary material for: Analysis of the hybrid genomes of two field isolates of the soil-borne fungal species Verticillium longisporum
Source: BMC Genomics. 2018 Jan 3;19:14. doi: 10.1186/s12864-017-4407-x (PMC5753508; doi:10.1186/s12864-017-4407-x)
Supplement: Supplementary file 13 — Distribution of predicted CAZy families. (PDF 64 kb) [file 12864_2017_4407_MOESM13_ESM.pdf]

Additional file 13: Distribution of predicted CAZy families.

Species	**GH**	**GT**	**CE**	**PL**	**CBM**	**AA**	**Total**
Verticillium longisporum - VL1	545	202	202	66	131	181	1327
Verticillium longisporum - VL2	516	165	181	63	133	174	1232
Verticillium dahliae	266	93	107	31	71	106	674
Verticillium albo-atrum	274	86	100	38	67	91	656
Alternaria brassicicola	226	89	105	24	53	90	587
Botrytis cinerea	243	99	107	11	54	83	597
Leptosphaeria maculans	218	95	99	20	44	85	561
Sclerotinia sclerotiorum	228	90	93	5	62	75	553

The CAZy families are: Glycosyl Hydrolases (GH), Glycosyl Transferases (GT), Carbohydrate Esterases (CE), Polysaccaride lysases (PL), Carbohydrate Binding Modules (CBM), Auxillary Activities (AA).
