# Supplementary material for: Analysis of the hybrid genomes of two field isolates of the soil-borne fungal species Verticillium longisporum
Source: BMC Genomics. 2018 Jan 3;19:14. doi: 10.1186/s12864-017-4407-x (PMC5753508; doi:10.1186/s12864-017-4407-x)
Supplement: Supplementary file 14 — Accession numbers for proteins used for mating gene phylogeny in Fig. 2. (PDF 112 kb) [file 12864_2017_4407_MOESM14_ESM.pdf]

Additional file 14: Accession numbers for proteins used for mating gene phylogeny in Figure 2.

**Abbreviation**	**Protein name**	**Domain**	**Organism**	**Accession number**
Va1	MAT-1	a1	*Verticillium albo-atrum*	XP_003007797
VL11a	Mat1-1-1	a1	*Verticillium longisporum*	CRK40698
VL21a	Mat1-1-1	a1	*Verticillium longisporum*	CRK15093
VL11b	Mat1-1-1	a1	*Verticillium longisporum*	CRK19038
Vd1	MAT1-1-1	a1	*Verticillium dahliae*	BAG83052
VL21b	Mat1-1-1	a1	*Verticillium longisporum*	CRK15107
Gf1	MAT-1-1	a1	*Gibberella fujikuroi*	AAC71055
Gz1	MAT1-1-1	a1	*Gibberella zeae*	AAG42809
Pm1	MAT1-1-1	a1	*Penicillium marneffei*	ABC68484.1
Ac1	MAT-1	a1	*Ajellomyces capsulatus*	ABO87596.1
Af1	MAT1-1-1	a1	*Aspergillus fumigatus*	AAX83123.1
An1	MAT-1	a1	*Aspergillus nidulans*	EAA63189.1
Rs1	MAT-1-1	a1	*Rhynchosporium secalis*	CAD71141.1
Pb1	MAT-1	a1	*Pyrenopeziza brassicae*	CAA06844
Pa1	FMR1	a1	*Podospora anserina*	S22448
Sm1	SMTA-1	a1	*Sordaria macrospora*	O42837
Nc1	MAT-1	a1	*Neurospora crassa*	AAC37478
Mg1	MAT1-1-1	a1	*Magnaporte grisea*	BAC65083.1
Zt1	MAT-1-1	a1	*Zymoseptoria tritici*	AF440399_2
Aa1	MAT-1	a1	*Alternaria alternata*	BAA75907.1
Ab1	MAT-1	a1	*Alternaria brassicicola*	AAK85542.1
Ss1	MAT-1	a1	*Stemphylium sarciniforme*	AAR04460
Ch1	MAT-1	a1	*Cochliobolus heterostrophus*	1913430A
Cp1	MAT1-1-1	a1	*Cryphonectria parasitica*	AF380365_3
Lt1	MAT-1	a1	*Lachancea thermotolerans*	XP_002554225.1
Zr1	MAT-1	a1	*Zygosaccharomyces rouxii*	XP_002497889.1
Sc1	MAT-1	a1	*Saccharomyces cerevisiae*	EDN62161.1
Ca1	MAT-1	a1	*Candida albicans*	XP_714749
Vd2	MAT1-2-1	MATA_HMG	*Verticillium dahliae*	BAG12301.1
Pa2a	MAT1-2-1/FPR1	MATA_HMG	*Podospora anserina*	CAA45520.1
Sm2	SMTa-1	MATA_HMG	*Sordaria macrospora*	CAA71624.1
Nc2a	MAT1-2-1/mat a-1	MATA_HMG	*Neurospora crassa*	AAA33598
Mg2	MAT1-2-1	MATA_HMG	*Magnaporte grisea*	BAC65094.1
Dp2	MAT-2	MATA_HMG	*Dothistroma pini*	ABK91353
Zt2	MAT1-2-1	MATA_HMG	*Zymoseptoria tritici*	AAL30836.1
Ac2	MAT-2	MATA_HMG	*Ajellomyces capsulatus*	EER39720.1
Pm2	MAT-2	MATA_HMG	*Penicillium marneffei*	XP_002151220.1
Af2	MAT-2	MATA_HMG	*Aspergillus fumigatus*	XP_751745.1
An2	MAT-2	MATA_HMG	*Aspergillus nidulans*	CBF85903.1
Pb6	SexP	SexP	*Phycomyces blakesleeanus*	ABX27912.1
VL22	MAT1-2-1	MATA_HMG	*Verticillium longisporum*	CRK15090
VL12	MAT1-2-1	MATA_HMG	*Verticillium longisporum*	CRK40699
Cp2	MAT1-1-3	MATA_HMG	*Cryphonectria parasitica*	AAK83344.1
Gf2	MAT1-1-3	MATA_HMG	*Gibberella fujikuroi*	AAC71053.1
Fg2	MAT1-1-3	MATA_HMG	*Fusarium acaciae-mearnsii*	ABE98373.1
Gz2	MAT1-1-3	MATA_HMG	*Gibberella zeae*	AAG42812
Pa2b	MAT1-1-3/SMR2	MATA_HMG	*Podospora anserina*	CAA52051
Nc2b	MAT1-1-3/mat	MATA_HMG	*Neurospora crassa*	AAC37476
Pt2	MAT-2	MATA_HMG	*Pyrenophora teres*	AAY35017
Bs2	MAT-2	MATA_HMG	*Bipolaris sacchari*	CAA65081.1
Ch2a	MAT1-2-1	MATA_HMG	*Cochliobolus heterostrophus*	CAA48464.1
Ch2b	MAT-2/1	MATA_HMG	*Cochliobolus homomorphus*	AAD33441.1
Aa2	MAT-2	MATA_HMG	*Alternaria alternata*	BAA75903.1
Ca2	Rfg1p	MATA_HMG	*Candida albicans*	XP_715804.1
Sc2	Rox1p	MATA_HMG	*Saccharomyces cerevisiae*	NP_015390.1
Tr3	SOX8b	SOX	*Takifugu rubripes*	AAQ18506
Cb3	SRY	SOX	*Cervus elaphus yarkandensis*	ABK91721
Mm3a	SOX-1	SOX	*Mus musculus*	NP_033259.2

Pb7	SexM	SexM	*Phycomyces blakesleeanus*	ABX27909.1
Pc2	STE11	MATA_HMG	*Pneumocystis carinii*	Q870J1
Sp2	STE11	MATA_HMG	*Schizosaccharomyces pombe*	CAA77507.1
Sj2	STE11	MATA_HMG	*Schizosaccharomyces japonicus*	XP_002175130
Um2	Prf1	MATA_HMG	*Ustilago maydis*	AAC32736
Pb2	MAT1-1-3/phb1	MATA_HMG	*Pyrenopeziza brassicae*	CAA06846.1
Cq3	pangolin	SOX	*Culex quinquefasciatus*	XP_001864781
Am3	hypothetical	SOX	*Ailuropoda melanoleuca*	EFB23328
Dm3a	pangolin	SOX	*Drosophila melanogaster*	NP_001014685.1
Dr3	TF-7	SOX	*Danio rerio*	AAI63927
Xl3	XTCF-3b	SOX	*Xenopus laevis*	CAA67689
Cs4	TF	HMGB	*Ciona savignyi*	NP_001071831.1
Sp3	Tcf/Lef	SOX	*Strongylocentrotus purpuratus*	NP_999640.1
Mm3b	lymphoid enhancer	SOX	*Mus musculus*	EDL12207.1
Mm3c	HMG	SOX	*Mus musculus*	NP_694878.2
Dm3b	bobby sox	SOX	*Drosophila melanogaster*	NP_001027087.1
Ag3	AGAP003896-PA	SOX	*Anopheles gambiae*	XP_001230616.1
Sc4a	NHP6A	HMGB	*Saccharomyces cerevisiae*	EDN61184
Ce4	HMG-4	HMGB	*Caenorhabditis elegans*	NP_498633
At4	HMG	HMGB	*Arabidopsis thaliana*	AAK43965.1
Al5	HMG	HMG	*Antonospora locustae*	ACI87876.1
Xl4a	TF1-B	HMGB	*Xenopus laevis*	NP_001079429
Dr4a	RNA Polymerase like	HMGB	*Danio rerio*	CAQ14015
Dr4b	TF	HMGB	*Danio rerio*	NP_957297
Hs4a	RNA Polymerase 1	HMGB	*Homo sapiens*	EAW51616
Xl4b	ubtf-b	HMGB	*Xenopus laevis*	AAH42232
Ec5	HMG	HMG	*Encephalitozoon cuniculi*	NP_585883
Eb5	chromatin-associated	HMG	*Enterocytozoon bieneusi*	XP_002652255.1
Bf4	HMG	HMGB	*Botryotinia fuckeliana*	XP_001548220
Sc4b	NHP6B	HMGB	*Saccharomyces cerevisiae*	NP_010459
Hs4b	HMG	HMGB	*Homo sapiens*	2CS1_A
Dr4c	TF	HMGB	*Danio rerio*	NP_957297.1
Hs4c	TF1	HMGB	*Homo sapiens*	NP_055048.1
Xl4c	ubtf-b	HMGB	*Xenopus laevis*	AAH42232.1
Sp5	Pc	HMG	*Schizosaccharomyces pombe*	P10841

a1= α1
